# Triclosan Resistome from Metagenome Reveals Diverse Enoyl Acyl Carrier Protein Reductases and Selective Enrichment of Triclosan Resistance Genes

**DOI:** 10.1038/srep32322

**Published:** 2016-08-31

**Authors:** Raees Khan, Hyun Gi Kong, Yong-Hoon Jung, Jinhee Choi, Kwang-Yeol Baek, Eul Chul Hwang, Seon-Woo Lee

**Affiliations:** 1 Department of Applied Biology, Dong-A University, Busan 604-714, Republic of Korea; 2 Department of Applied Bioscience, Dong-A University, Busan 604-714, Republic of Korea

## Abstract

Triclosan (TCS) is a widely used antimicrobial agent and TCS resistance is considered to have evolved in diverse organisms with extensive use of TCS, but distribution of TCS resistance has not been well characterized. Functional screening of the soil metagenome in this study has revealed that a variety of target enoyl acyl carrier protein reductases (ENR) homologues are responsible for the majority of TCS resistance. Diverse ENRs similar to 7-α-hydroxysteroid dehydrogenase (7-α-HSDH), FabG, or the unusual YX7K-type ENR conferred extreme tolerance to TCS. The TCS-refractory 7-α HSDH-like ENR and the TCS-resistant YX7K-type ENR seem to be prevalent in human pathogenic bacteria, suggesting that a selective enrichment occurred in pathogenic bacteria in soil. Additionally, resistance to multiple antibiotics was found to be mediated by antibiotic resistance genes that co-localize with TCS resistance determinants. Further comparative analysis of ENRs from 13 different environments has revealed a huge diversity of both prototypic and metagenomic TCS-resistant ENRs, in addition to a selective enrichment of TCS-resistant specific ENRs in presumably TCS-contaminated environments with reduced ENR diversity. Our results suggest that long-term extensive use of TCS can lead to the selective emergence of TCS-resistant bacterial pathogens, possibly with additional resistance to multiple antibiotics, in natural environments.

The emergence of bacterial resistance to antimicrobials and the growing number of multidrug-resistant bacteria have become a global public health concern^1^ and have precipitated the need for the development of more effective antibiotics^2^. It is widely accepted that the development and spread of antibiotic resistance in microbes can be largely attributed to the abuse and misuse of antibiotics and biocides^3^. A direct correlation between biocide and antimicrobial use and the extent of antimicrobial resistance has been demonstrated^3^,^4^.

Various environments, including waste water treatment plants (WWTPs), sediment, surface water, sewage, sludge and soil, have been known to serve as potential reservoirs of antibiotic resistance genes (ARGs)^5^. Exchange of ARGs by horizontal gene transfer between bacteria from natural environments and pathogenic bacteria has been reported^6^. This phenomenon reveals the importance of the environmental resistome in terms of the possible transmission and spread of selected ARGs to human pathogenic bacteria.

The widely used biocide triclosan [5-chloro-2-(2,4-dichlorophenoxy)-phenol, TCS] has a broad-spectrum capacity to kill many microorganisms and is a constituent in a variety of personal care products^7^. TCS blocks bacterial type II fatty acid synthesis by targeting enoyl-acyl carrier protein reductases (ENRs), which catalyze the last enoyl reduction step in the fatty acid elongation cycle^8^. So far, four ENR isozymes have been reported in bacteria, namely FabI, FabL, FabV, and FabK^9^. FabI and FabL ENRs are known to share a similar YX6K type catalytic domain, whereas FabV carry a YX8K type catalytic domain^10^. All ENRs are members of the short-chain dehydrogenase/reductase (SDR) superfamily, except for FabK, which is completely TCS-refractory and is a non-SDR ENR^9^.

In addition to being targeted by TCS, ENRs are also targeted by a variety of other clinically important antibiotics and antimicrobials, such as Isoniazid^11^ the soon to be marketed AFN-1252^12^, hexachlorophene, diflufenican^13^,^14^ and various other synthetic and natural antimicrobials^11^,^13^. Some mutations in the active sites of ENRs, such as the substrate- and cofactor-binding domains, lead to tolerance of the ENR inhibitors^14^,^15^. The altered ENRs undergo subsequent conformational changes that seem to be the major resistance mechanism against TCS and related antimicrobials^16^.

TCS-resistant bacteria are abundant in nature. Several mechanisms that confer resistance to this biocide are known and include: (i) overexpression of ENR^17^; (ii) presence of mutated and/or TCS-tolerant ENR^18^; (iii) changes in the outer membrane^19^; (iv) up regulation of efflux pumps^17^ and (v) the presence of other potential target genes^20^. Additionally, TCS has been reported to pose selective pressure^21^ and to induce cross-resistance to other antibiotics in various microorganisms^18^,^22^.

An enormous amount of TCS has been released into the environment^23^; therefore, the microorganisms living in TCS-contaminated environments may have evolved certain mechanisms to cope with the presence of this biocide. Limited knowledge exists about TCS resistance in a large number of uncultured microorganisms, which constitute pools of resistance determinants potentially transferrable to human pathogens. In this study, we primarily aimed to (1) investigate the TCS resistome from the soil metagenome using functional metagenomics-based screening, (2) to investigate any resistance to other antibiotics conferred by ARGs colocalized with TCS resistance determinants, (3) to perform comprehensive profiling of both prototypic and TCS-resistant metagenomic ENR diversity from different environments, and (4) to assess selective specific ENR abundance in presumably TCS-rich environments.

## Results

### Selection of TCS-resistant clones

Metagenomic libraries with the average insert DNA size of 35 kb, representing approximately 4.34 Gb of metagenomic DNA (equivalent to approximately 936 *Escherichia coli* K-12 genomes) from alluvial soil (AS) and industrially contaminated soil (ICS) (Supplementary Table S1), were screened for TCS resistance. AS was collected from the sediments of Nakdong river water in the Eulsukdo island area, where TCS was detected at approximately 0.66 μg/L (data not shown). The AS receives combined sewer overflow from different sites, including the Sasang-gu industrial complex, where ICS was collected. TCS was detected at approximately 1.07 μg/kg and 1.29 μg/kg in AS and ICS samples, respectively (data not shown). A total of 123 fosmid clones with TCS resistance were classified into 32 groups (Table 1) on the basis of their restriction digestion profiles (data not shown). Shotgun library construction and transposon-based mutagenesis of those 32 representative clones were conducted, followed by sequence analysis to identify candidate genes responsible for TCS resistance.

### Metagenomic clones carried diverse TCS-resistance determinants

**Figure 1:**
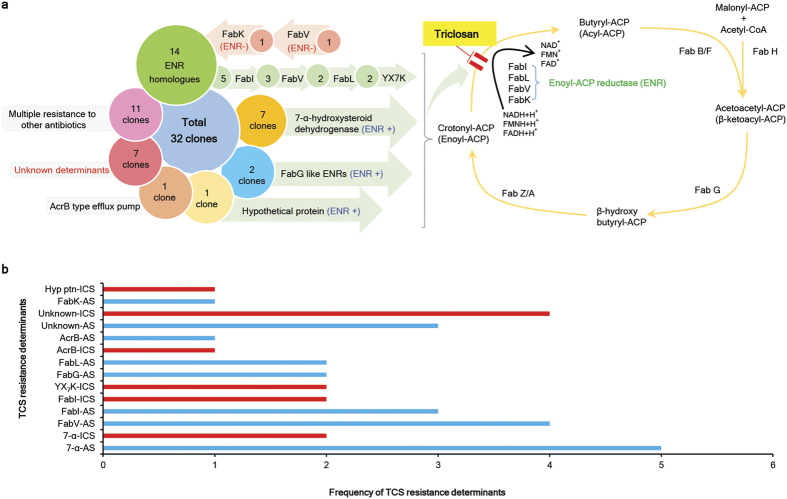
Metagenomic analysis of TCS-resistant clones revealed diverse patterns of TCS resistance determinants, with a higher diversity observed in AS compared to ICS. (**a**) A summarized sketch of the TCS resistance determinants from metagenomic library screening. The majority of the clones carried different versions of ENR homologues, including previously known prototypic ENRs, novel candidate ENRs, and ENR homologues with compromised ENR activity. The ENRs that complemented ENR activity *in vivo* are indicated by forward light green arrows or are indicated otherwise. Other clones carried TCS resistance efflux pumps, hypothetical proteins or unknown determinants for resistance to this biocide. Some of the clones even carried genes for resistance to other antibiotics colocalized with the TCS resistance determinants. (**b**) Distribution of TCS resistance determinants between AS and ICS. TCS resistance determinants in AS and ICS are indicated in cyan and red, respectively. The comparative diversity of TCS resistance determinants was higher in AS compared to ICS.

Metagenome-derived TCS-resistance determinants identified in this study fell into several different groups. The majority of the clones either carried different versions of ENRs, including prototypic FabI, FabV, FabK or FabL-like ENR homologues, or novel candidate 7-α-HSDH, FabG and YX7K-like ENR homologues (Fig. 1a). Other clones carried efflux pumps, novel hypothetical proteins, or unknown determinants that conferred resistance to TCS. ENR or FabG activity of selected metagenomic clones was confirmed by genetic complementation of temperature-sensitive (ts) *fabI* or *fabG* mutants of *E. coli*, respectively (Table 1). A higher diversity of TCS resistance determinants was observed in AS compared to ICS (Fig. 1b).

**Figure 2:**
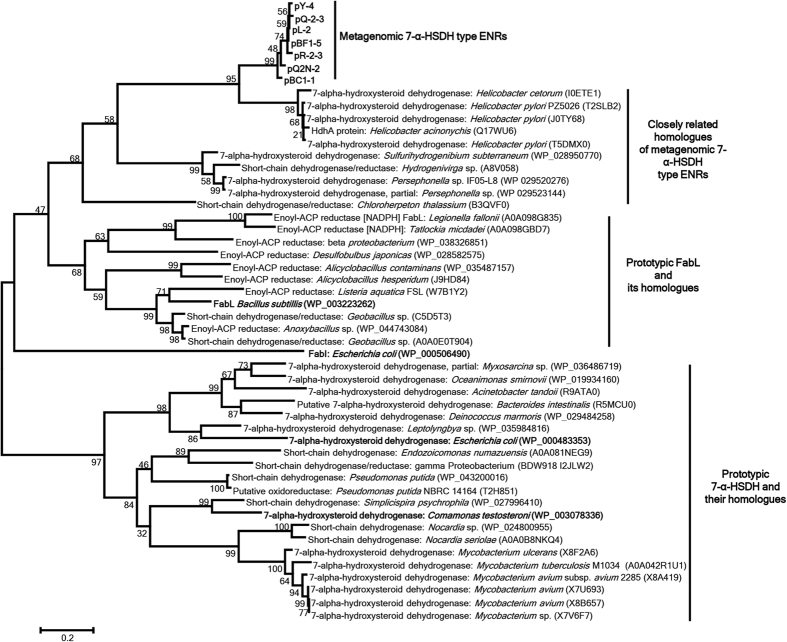
The metagenomic 7-α-HSDH-type ENRs and their homologues clustered as a separate clade from other closely related prototypic ENRs. Phylogenetic tree of metagenomic 7-α-HSDH-type ENRs with prototypic 7-α-HSDH, FabL and FabI (in bold). Maximum likelihood analysis of well-characterized 7-α-HSDH, FabL and FabI enzymes (in bold) and their homologues with sequence identity over 50% from the Uniref50 database. Bootstrap values are indicated for each node in a bootstrap analysis of 500 replicates. The scale bar represents 0.2 estimated amino acid substitutions per residue.

RAIphy (relative abundance index phylogenetic analysis)-based taxonomic assignment of metagenomic clones revealed that the majority belonged to the phylum proteobacteria (Supplementary Table S2). The metagenomic 7-α-HSDH-type ENRs and their closest homologues (obtained from the Uniref50 database) clustered together in the FabL clade, but the well characterized 7-α-HSDH from *E. coli* and *C. testosteroni* clustered together on a separate clade (Fig. 2). Both the YX7K and FabG-type metagenomic ENR homologues clustered on a separate clade (Supplementary Fig. S1a,b)

### Metagenome-derived TCS resistance-associated FabI ENR substitutions were predominant in human-associated pathogenic bacteria and soil-borne plant pathogens

**Figure 3:**
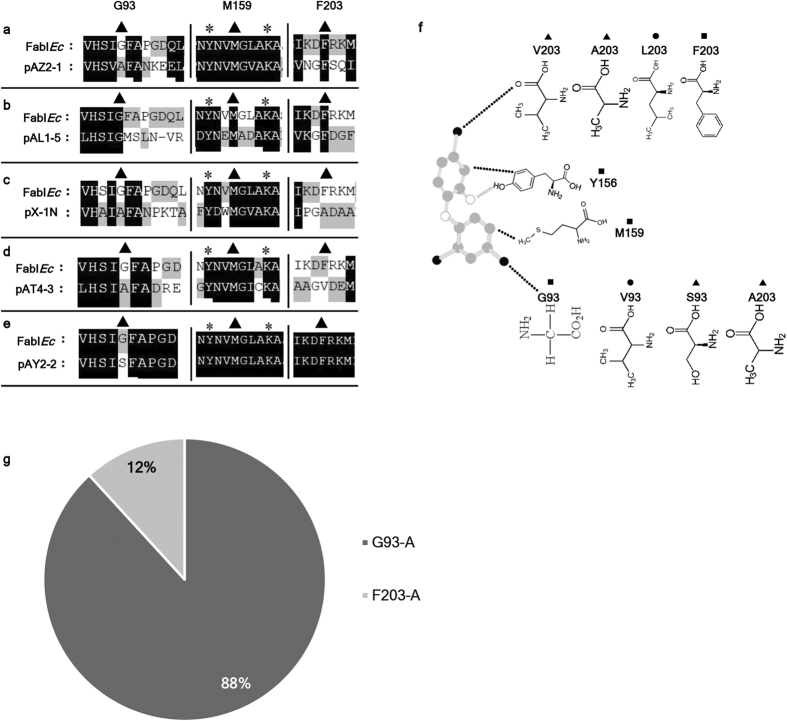
Metagenomic TCS-resistant FabI ENR homologues revealed unique patterns of substitutions that were abundant in most of the human-associated pathogenic and nonpathogenic bacterial FabI ENR homologues. (**a–e**) Multiple alignment of metagenomic FabI ENR homologues with *E. coli* FabI ENR (on top). Only the multiple alignment regions carrying substitutuions associated with TCS resistance are presented here. Additionally, each original alignment has been divided into three sections, each separated by a vertical line, with individual alignments for each different ENR separated by horizontal lines. The YX6K-type active sites are marked with asterisks. Important residues whose mutations are associated with TCS resistance are indicated with closed triangles. (**f**) Schematic drawings of TCS interactions with important residues of the FabI ENR that are associated with TCS resistance. Ball-and-stick representation of TCS, with gray, black, and open white balls indicating carbon, chloride, and oxygen, respectively. Hydrogen bonds between TCS and amino acids are indicated by open white dotted lines, and hydrophobic interactions are indicated by black dotted lines. The neutral and mutated residues in the case of prototypic FabI are indicated with black rectangles and black circles, respectively. Amino acid substitutions found in the metagenomic ENRs are indicated with black triangles. Metagenomic TCS resistance associated amino acid substitutions carried different side chains. The variation in amino acid side chains at these positions are previously reported to be associated with triclosan tolerance^16^. (**g**) Metagenome-derived TCS-resistance-associated substitutions were dominant in FabI homologues of 401 pathogenic and non-pathogenic bacterial strains (122 bacterial species) (Supplementary Data 1). The G_93_-A substitution was more commonly present in 50% of bacterial ENRs, followed by the F_203_-A substitution, which was present in 7% of the bacterial ENRs from the investigated 401 bacterial strains. Therefore, the G_93_-A and F_203_-A substitutions constitute 88% and 12% of those found in ENRs, respectively.

**Figure 4:**
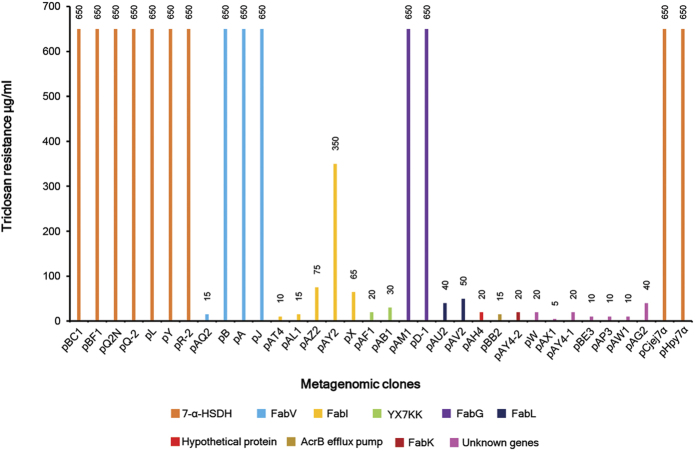
Metagenomic clones revealed various levels of TCS resistance. The levels of TCS resistance of all metagenomic clones and clones carrying metagenomic ENR homologues from *Helicobacter pylori* HPKTCC B0100 and *Campylobacter jejuni* NCTC11168 were determined for all metagenomic clones up to the maximum level of 650 μg/ml TCS. TCS-refractory clones carrying homologues of 7-α-HSDH, FabG and FabV ENR exhibited the maximum level of resistance, 650 μg/ml TCS. Different candidate genes responsible for TCS resistance in the metagenomic clones are indicated by differently colored bars, or as otherwise indicated.

Among the various metagenome-derived and TCS-resistant ENR homologues, five were FabI homologues (Table 1) carrying typical YX6K-type catalytic domains (Fig. 3a–e). These FabI ENR homologues conferred various levels of resistance to TCS (Fig. 4, Supplementary Table S3). Mutations that are well known to confer TCS resistance to the *E. coli* ENR FabI and to the *Mycobacterium* ENR InhA include amino acid substitutions such as G_93_-V, G_93_-S, M_159_-T, F_203_-L and F_203_-C. However, the metagenomic FabI ENRs carried unique patterns of substitutions (Fig. 3a–e), such as G_93_-A, F_203_-A and F_203_-V, which differed from the previously known amino acid substitutions^15^,^24^. Additionally, these residues in the metagenomic FabI ENRs carry different side chains, which in turn may affect protein-TCS interactions (Fig. 3f). We performed genome-wide investigations of FabI ENR variations to examine the comparative abundance of metagenomic-identified substitutions and previously known FabI ENR mutations in human-associated pathogenic bacteria, non-pathogenic bacteria and soil-borne plant pathogenic bacteria (Supplementary Data 1). Most of the FabI ENR homologues from these organisms carried substitutions similar to those found in the metagenomic ENRs rather than mutations reported in the previously known FabI ENRs of *E. coli* and other organisms (Fig. 3g, Supplementary Data 1). Metagenomic FabI ENR substitutions were mostly present as single point mutations (G_93_-A or F_203_-A) in the FabI ENRs of these organisms. Among these substitutions, the frequency of G_93_-A replacement was higher (50%) than F_203_-A (7%) in ENRs from these organisms. Few of the FabI ENR homologues carried substitutions, which were different from both previously known and metagenome-derived substitutions. Additionally, the metagenome-derived FabI ENRs, with different patterns of substitutions, not only conferred parental levels of resistance to TCS but also complemented ENR activity (Table 1).

### Metagenomic YX7K-type ENRs shared by both eukaryotes and prokaryotes are abundant among intracellular pathogenic bacteria and apicomplexans

Two of the metagenomic subclones, pAB1-3 and pAF1-6 (Table 1) carried intermediate TCS-resistant ENR homologues, (Fig. 4, Supplementary Table S3) that shared the highest identity with the ENR from *Chlamydia trachomatis*^25^, which was recently reported to be essential for the pathogenicity of this organism. Comparison of these ENRs with known bacterial prototypic ENRs revealed that the metagenomic and closely related ENRs from *C. trachomatis* not only carried different catalytic domains from the YX7K type and additional loops in their structure (Supplementary Fig. S2a,b) but also shared the least identity with other prototypic ENRs. These YX7K-type ENRs were abundant in many obligate intracellular pathogenic bacteria and apicomplexan microorganisms (Supplementary Table S4–S5), where the YX7K-type catalytic domain was highly conserved, along with other shared features.

### TCS-refractory 7-α-HSDH-like ENR homologues from the metagenome are predominant in Epsilonproteobacteria

Seven of the completely TCS-refractory metagenomic clones, pBF1-5, pL-2, pY-4, pR-2-3, pQ-2-3, pQ2N-2 and pBC1-1 (Fig. 4, Supplementary Table S3, Table 1) carried genes encoding 7-α-HSDH homologues that conferred a high level of TCS resistance to TCS-sensitive *E. coli*, increasing the minimal inhibitory concentration (MIC) of this biocide by >700-fold. These metagenomic 7-α-HSDH homologues also shared structural similarity with some of the previously known prototypic ENRs (Supplementary Fig. S3a) and indeed complemented a ts-*fabI* ENR mutant of *E. coli*, confirming its ENR activity (Table 1). However, the 7-α-HSDH homologues were highly identical (96–69%) to 7-α-HSDH from members of *Epsilonproteobacteria*, most of which are human pathogens, such as *Arcobacter, Campylobacter* and *Helicobacter* species (Supplementary Table S6). These metagenomic ENR homologues share conserved catalytic amino acids with the well-characterized 7-α-HSDH from *E. coli* and *C. testosteroni* (Supplementary Fig. S3b). However, the catalytic domains of the metagenomic 7-α-HSDH-like ENR homologues differed from that of *E. coli* and *C. testosteroni* by replacement of Y_159_/Y_161_ with histidine (compared with *E. coli* and *C. testosteroni* 7-α-HSDH, respectively).

### The 7-α-HSDH homologues of *Helicobacter pylori* and *Campylobacter jejuni* confer similar levels of resistance to TCS as metagenomic 7-α-HSDH

Metagenome-derived 7-α-HSDH gene homologues were predominant in three major families of *Epsilonproteobacteria* – *Campylobacteraceae*, *Helicobacteraceae* and *Nautiliaceae –* the first two of which include many human and other animal pathogens (Supplementary Table S6). In fact, the 7-α-HSDH gene homologues of *C. jejuni* NCTC11168 and *H. pylori* HPKTCC B0100 were able to confer the equivalent MIC to TCS in naturally TCS-sensitive *E. coli* (Fig. 4). Comparison of the 7-α-HSDH gene homologues from *C. jejuni* and *H. pylori* with closely related and well characterized prototypic 7-α-HSDH and ENRs revealed that these enzymes shared similar key enzyme features (Supplementary Fig. S3c,d). Furthermore, the 7-α-HSDH homologues from *C. jejuni* and *H. pylori* exhibited enoyl-reductase activity by successfully complementing a ts-*fabI* mutation of *E. coli* (Table 1).

### 3-oxoacyl-ACP reductase (FabG)-like ENRs with compromised FabG activity confer significant resistance to TCS

Two TCS-refractory clones, pD-1-7 and pAM1-4 (Fig. 4) carried a gene encoding FabG for TCS resistance (Table 1, Supplementary Table S3). These metagenomic FabG homologues shared weak similarity with the FabL ENR from *Bacillus subtilis* of the prototypic ENRs, and FabG-like ENR homologues were also able to be found from *Streptomyces* and other bacterial species through our genome-wide similarity search (Supplementary Table S7). Both metagenomic FabG-like ENR candidates and the well-characterized FabG from *E. coli* displayed the presence of similar key enzyme features (Supplementary Fig. S4). However, the catalytic triad (Ser_138_, Tyr_151,_ and Lys_155_) of the metagenomic FabG-like ENR candidates differed from the functional FabG of *E. coli* by the replacement of Tyr_151_ with a nonpolar valine. While metagenomic FabG-like ENRs did not carry the highly conserved Asn_113_, a characteristic of the FabL ENRs, these two homologues complemented the growth of a FabI-defective *E. coli* JP1111 mutant (Table 1). However, the metagenomic FabG homologues were unable to complement the original FabG activity in the *E. coli* temperature-sensitive *fabG* mutant CL37, suggesting that an ENR that is highly similar to FabG is present.

### Metagenomic clones with TCS-resistant prototypic FabV, FabL, and FabK-type ENR homologues have various patterns of TCS resistance

Seven of the metagenomic clones carried prototypic FabV, FabL and FabK-like ENR homologues (Table 1), which conferred various levels of TCS resistance (Fig. 4, Supplementary Table S3). Unlike other metagenomic FabV ENRs, the pAQ2 FabV ENR lacked the conserved FAD-binding domain (Supplementary Fig. S5a), showed lower resistance (Fig. 4) and had a compromised ENR activity. Two FabL gene homologues from the metagenomic clones (Table 1) complemented the ENR activity (Table 1), conferred resistance to TCS (Supplementary Table S3), and exhibited key features of FabL, including having a YX6K-type catalytic domain (Supplementary Fig. S5b). One of the metagenomic clones carried a FabK ENR homologue that was mildly TCS-resistant, unlike other prototypic FabK ENRs, and its ENR activity was compromised (Table 1, Fig. 4). Comparison with a prototypic FabK ENR from *S. pneumonia* revealed that the metagenome-derived FabK homologue lacks the conserved His_144_ residue, which is known to be involved in the catalytic activity of FabK-like ENRs (Supplementary Fig. S5c).

### TCS resistance was mediated by a novel hypothetical-protein-homologue-like ENR candidate and non-ENR genes in some metagenomic clones

Nine of the TCS-resistant metagenome-derived clones did not carry any previously known ENR homologues (Supplementary Table S8, Supplementary Fig. S6a–i/Supplementary Table S9–S17). An *acrB* gene homologue (Supplementary Fig. S6h, Supplementary Table S16) and a gene encoding a novel hypothetical protein homologue (which complemented ENR activity *in vivo*) (Table 1, Supplementary Fig. S6i, Supplementary Table S17) were also responsible for TCS resistance. Inactivation of AcrB or the hypothetical-protein-like ENR homologue by Tn insertion led to the complete loss of TCS resistance. Intriguingly, a variety of microorganisms, including the potential human pathogen *Massilia timonae*, contained a metagenome-derived pAH4-3 hypothetical-protein-like homologue (Supplementary Table S18). The remaining seven clones did not carry any genes previously known to confer resistance to TCS (Supplementary Table S8), although some of them carried different efflux pump protein homologues. In addition, none of the seven *E. coli* clones exhibited any significant decrease in TCS concentration in the medium (data not shown), suggesting that TCS is not being removed or inactivated by those seven clones, nor is ENR activity complemented in those clones.

### Metagenomic TCS-resistant clones conferred co/cross-resistance to other antibiotics

Eleven of the TCS-resistant clones showed resistance to at least one of the antibiotics tested (Supplementary Table S19–S20, Supplementary Fig. S6a,c,e,i, Supplementary Fig. S7a–g, Supplementary Tables S9, S11, S13, S17, S21–S27). The pBC1 clone, which carried two different homologues of the *acrB* gene (Supplementary Fig. S7g, Supplementary Table S27), showed mild cross-resistance to tetracycline when treated with sub-lethal concentrations of TCS. The pAV2 clone, which showed co-resistance against other antibiotics, contained an ARG cluster colocalized with the TCS-resistant ENR (Supplementary Fig. S7c, Supplementary Table S23). This cluster contained genes encoding two multidrug efflux pump family protein homologues and an aminoglycoside-modifying enzyme, along with a TCS-resistant ENR homologue. Most of the metagenomic clones that showed co/cross-resistance to TCS contained colocalized genes that conferred resistance to other antibiotics, either in the form of efflux pumps or antibiotic-modifying enzymes (Supplementary Table S28). However, two clones did not carry any genes known for conferring antibiotic resistance, even though both showed co-resistance to other antibiotics. Furthermore, seven of the metagenomic clones contained mobile genetic element signatures (Supplementary Table S29). The accession numbers for the nucleotide sequences of the metagenomic TCS resistance clones/subclones are presented in Supplementary Table S30.

### Metagenomic and prototypic ENRs are abundant in nature, and presumably TCS-rich environments (TCSRE) tend to enrich for TCS-tolerant ENRs

**Figure 5:**
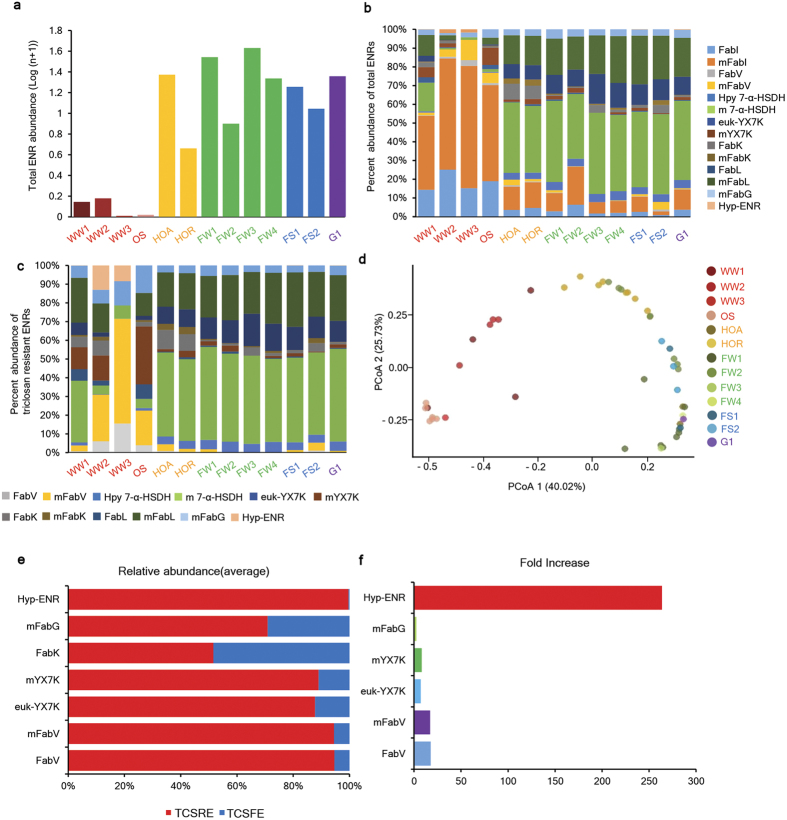
Presumably TCSREs tend to shape ENR diversity and abundance profiles in a different way than the presumably TCS-free or low-TCS environments. The ‘m’ preceding each ENR denotes its metagenome origin. (**a**) Total ENR abundance. Total ENR abundance was significantly lower in presumably TCSRE. Presumably TCS-free environments (TCSFE) showed a high abundance of ENRs. WW1, waste water-WWTP-China; WW2, activated sludge-WWTP China; WW3, waste water-WWTP Hong Kong; OS, Ocean sediment-China; HOA, ancient human oral-Archeological site; HOR, human oral-Spain; FW1, fresh water-Antarctica; FW2, fresh water-Minnesota; FW3, fresh water-Australia; FW4, fresh water-South Australia; FS1, Mediterranean forest-Spain; FS2, cave-temperate forest-Finland; G1, glacier-Austria. (**b**) Percent abundances of total ENRs. Total ENR abundances revealed different diversity patterns for presumably TCS-rich environments. The TCSFEs tended to have similar ENR diversity patterns that were distinct from those of TCSREs. Each color represents a unique ENR subtype. FabI and mFabI were the major ENRs in TCSREs, whereas the metagenome-derived 7-α-HSDH type ENRs were predominant in TCSFEs. (**c**) relative abundances of the TCS-tolerant ENRs. The percentage of TCS-tolerant ENRs was expressed as the number of TCS-tolerant genes divided by the total number of TCS-tolerant ENR reads. TCSREs tended to have similar TCS-tolerant ENR diversity profiles that were distinct from those of TCSFEs. FabV-type ENRs and other TCS-tolerant ENRs, such as the metagenome-derived YX7K-type ENR, the metagenome-derived FabG-like ENR homologues, and the novel hypothetical-protein-like ENR homologues, were comparatively enriched in TCSREs. (**d**) Principal coordinate analysis (PCoA) plot based on ENR relative abundances showing the ENR composition differences among 49 environmental samples that included ENRs from TCS-free or low-TCS environments. Each point represents a metagenomic sample, and colors denote the region from which the metagenome sample originated. The variance explained by the PCoA is indicated on the axes. (**e**) Relative abundance of selectively enriched TCS-tolerant ENRs in TCSREs and TCSFEs. Hypothetical-protein-like ENR, mFabG, FabK, mYX7K, euk-YX7K, mFabV and FabV-like ENR homologues were relatively abundant in TCSREs. (**f**) Fold increases of TCS-tolerant ENRs in TCSREs. Multifold increases in hypothetical-protein-like ENR, mFabG, mYX7K, euk-YX7K, mFabV and FabV-like ENR homologues were observed.

An environmental survey was performed using 49 environmental shotgun metagenomic DNA data sets, representing approximately 26.6 Gb of metagenomic DNA (equivalent to approximately 5740 *E. coli* K-12 genomes) obtained from MG-RAST, a public-repository web site (Supplementary Data 2). These metagenomic datasets cover 13 different environments, and the WWTPs and ocean sediment were considered TCSREs relative to the presumed TCS-free environments (TCSFEs) or low TCS environments, such as the glacier, cave, forest, fresh water and human oral cavity environments. These metagenomic datasets were screened for the presence of both metagenomic and prototypic ENR homologues. Total ENR abundance analysis revealed that ENR content in TCSREs was greatly reduced compared to TCSFEs (Fig. 5a). A comparative analysis for both TCS-sensitive (prototypic) and TCS-resistant (prototypic and metagenomics) ENRs showed that TCSREs tended to shape the ENR diversity in a different way compared to TCSFEs (Fig. 5b). The TCSFEs were more likely to share similar ENR diversity patterns. Interestingly, the metagenome-derived 7-α-HSDH-like ENR homologues were the most abundant ENRs (34–42%) in TCSFEs (Fig. 5b). A comparative abundance analysis for TCS-resistant ENRs revealed that TCSREs tended to enrich for TCS-tolerant prototypic (approximately 17.6-fold) and metagenomic (approximately 17.1-fold) FabV-type ENRs and other TCS-tolerant ENRs, such as the metagenome-derived YX7K-like (approximately 8.1-fold) and the metagenome-derived FabG-like (approximately 2.4-fold) ENR homologues (Fig. 5c,e,f). Interestingly, the metagenomic novel-hypothetical-protein-like ENR homologues were exceptionally enriched (approximately 263-fold) in TCSREs (Fig. 5c,e,f). Moreover, β-diversity analysis revealed that the ENR diversity in TCSREs was dissimilar from that found in TCSFEs (Fig. 5d).

## Discussion

Our results provide valuable information about the TCS resistance gene (TRG) reservoir in the natural environment at the metagenomic level. The level of TCS detected in our samples suggested potential TCS contamination of these sites, and is in concordance with various other studies^23^, where different TCS concentrations ranging from 0.02–35 μg/kg have been reported. Here, we demonstrate that novel metagenome-derived diverse ENR variants associated with TCS resistance are abundant in natural soils and in a number of human-associated pathogenic microorganisms. We submit that the excessive use of TCS and the frequent exposure of microorganisms to TCS may have led to modifications in ENR, the target enzyme, to the generation of various versions of functional redundancy in ENR activity. The prevalence of TCS-resistant FabI ENR homologues, which were abundant in most of the human pathogens investigated raises concerns about the efficacy of using TCS and TCS-based analogues against these microorganisms.

Intriguingly, our results revealed for the first time that a similar YX7K-type ENR is shared by both eukaryotic and prokaryotic intracellular pathogenic microorganisms, suggesting either a potential evolutionary link in the ENRs among these intracellular pathogens or a potential horizontal gene transfer event among these intracellular pathogenic organisms. Potential ENR dissemination via horizontal gene transfer have previously been assessed, where TCS resistance in a *Staphylococcus aureus* isolate was mediated by an unusual additional *sh-fabI* allele originated from and 100% identical to that of *S. haemolyticus*^26^. Additionally in other staphylococci, including *S. aureus* and *S. epidermidis* identical to *sh-fabI* homologues were located on plasmids, which suggested high mobility potential of these ENR homologues^26^. The metagenome-derived TCS-resistant 7-α-HSDH homologues were predominant in Epsilonproteobacteria; however, the lack of other prototypic ENR homologues in some of the members of this class, such as *Campylobacter lari* and *Helicobacter bilis,* indicate that 7-α-HSDH is the only ENR in these organisms. FabG-like and completely TCS-refractory ENRs were predominant in *Streptomyces* spp., which are known for having various polyketide synthesis pathways and might therefore have different types of ENRs for fatty acid biosynthesis. The slight structural similarities and differences in TCS resistance levels between metagenomic FabG and prototypic FabL ENRs suggest the possibility that enzymes of intermediate structures may have, over evolutionary history, left different versions of these enzymes throughout a diverse range of microorganisms. For instance, FabL and FabI are structurally very similar, with similar catalytic domains, but both have different cofactor requirements and different affinities for TCS. Additionally, phylogenetic analysis suggests that the metagenomic 7-α-HSDH, FabG and YX7K-like ENRs may have potentially diverged from FabL or FabI-like ENRs or from either 7-α-HSDH or FabG. Consequently, these different types of ENRs may be difficult to distinguish by sequence comparison or sequence annotation alone.

The unusual characteristics of two of the metagenomic prototypic FabV and FabK-like ENR homologues might be attributed to the absence of the conserved FAD-binding domain^27^ and the mutation in the catalytically active residue^28^, respectively. We assume that these nonfunctional ENRs may act as biocide load reducers by binding and sequestering a certain amount of TCS, thereby reducing the overall biocide burden of the functional ENR in bacterial cells. Our results suggested that even minute variations among these ENR homologues might result in conformational changes that, in turn, lead to the loss of ENR activity. To the best of our knowledge, this report is the first describing nonfunctional FabV and FabK ENR homologues with moderate TCS resistance.

It has been proposed that additional cellular targets of TCS might exist in addition to the previously known target^20^. TCS resistance conferred by the metagenomic partial *acrB* gene homologue revealed the presence of different versions of this protein or different codon usage^29^. Similarly, the novel hypothetical protein-encoding gene homologue from clone pAH4 and the unknown TCS resistance determinants in 7 other metagenomic clones further strengthen the hypothesis that novel ENRs and genes or efflux pumps can confer TCS resistance.

The co/cross-resistance to other antibiotics observed in the metagenomic clones may be associated with the colocalized genes encoding efflux pumps, antibiotic-modifying enzymes, ARG clusters and other genes. The ARGs that colocalized with TCS resistance determinants indicate that TCS may selectively enrich the complex patterns of multiple resistance to other antibiotics in the environment. TCS-mediated overexpression of the AcrB efflux system^22^,^30^ and the resulting co/cross-resistance to other classes of antibiotics have been extensively addressed^31^,^32^; however, our results provide clear evidence of co- or cross-resistance mediated not only by different versions of previously reported efflux pumps but also by other ARGs that colocalized with TCS resistance determinants.

Metagenomic comparisons from different environments suggest that TCS may act as a driving force to influence the total ENR diversity in TCSREs, which are the most likely sites for TCS resistance development^32^. Moreover, both the prototypic and metagenome-derived ENRs were abundant in nature, while one of the metagenomic 7-α-HSDH-type ENRs represented the majority of ENRs present in TCSFEs. The reduction in total ENR diversity, the similar diversity patterns and the selective enrichment of prototypic and metagenome-derived TCS-tolerant ENRs in TCSREs indicate that this biocide may pose a selective pressure for the enrichment of specific groups of bacteria carrying TCS-tolerant ENRs.

We conclude that different versions of ENRs and novel TCS resistant genes (TRGs) are abundant in nature. The vast diversity of ARG pools has made it possible for only some of the ARGs to be selectively transferred to many bacteria^8^,^33^; therefore, selective enrichment of TCS resistance in TCSREs^32^ and the spread of specific TRGs are possible in a diverse range of environmental and pathogenic microorganisms. The metagenome-derived novel patterns of TCS resistance raise concerns about the efficacy of this biocide and about the development of TCS- and non-TCS-based ENR inhibitors. The spread of these ENRs and any colocalized ARGs may lead to resistance to TCS, other ENR inhibitors, and multiple other antibiotics. Additionally, TCS may pose a selective pressure to enrich human- and soil-borne plant pathogenic bacteria for those with TCS resistance determinants in the environment. Moreover, the great diversity of the metagenome-derived ENR isozymes may provide a clue as to the evolution of different fatty acid synthesis systems in microorganisms and brings into question the evolutionary pressures that natural or synthetic inhibitors may have exerted in selecting for ENR diversity.

## Methods

### Bacterial strains, plasmids, genomic DNA and culture conditions

The bacterial strains and plasmids used in this study are described in Supplementary Table S31. Genomic DNA from *Helicobacter pylori* HPKTCC B0100 was obtained from the Korean Type Culture Collection (KTCC), Korea National Research Resource Bank (KNRRC). The genomic DNA from *Campylobacter jejuni* NCTC11168 was kindly provided by Dr. Jong-Hyun Kim at the Centers for Disease Control and Prevention. *E. coli* strains/mutants DH5α, EPI-300, JP1111 (obtained from *E. coli* genetic resources at Yale; CGSC) and CL37 (kindly provided by Dr. John Cronan at the University of Illinois at Urbana-Champaign) were routinely grown at 37 °C in Luria-Bertani (LB) broth or LB agar supplemented with appropriate antibiotics. The antibiotic concentrations used were as follows; ampicillin, 100 μg/ml; kanamycin, 50 μg/ml; chloramphenicol, 30 μg/ml and TCS, 0.1–650 μg/ml. Fosmid pCC1FOS (Epicentre Biotechnologies, Madison, WI, USA) was used to construct genomic libraries, whereas pUC119 and pGEM®-T Easy were used for further subcloning experiments.

### Determination of minimum inhibitory concentration (MIC)

To determine the MIC of TCS, *E. coli* EPI-300 with pCC1FOS and DH5α with pUC119 were first grown to an OD_600_ of 1.0, and these bacterial suspensions were serially diluted to 1 × 10^5^ colony-forming units (CFU)/ml. The cell suspension (1 × 10^5^ CFU/ml) was spread onto LB agar medium containing antibiotics and TCS in a range of 0.1–5 μg/ml. The LB plates were incubated at 37 °C for three days, and bacterial colony formation was examined at regular 24 h intervals. The lowest concentration of TCS (0.9 μg/ml) that prevented bacterial growth of *E. coli* EPI-300 was considered the MIC for TCS. To determine the MICs for the clones carrying various ENR homologues, the procedure was followed as above, but the negative control *E. coli* EPI-300 carried an additional chromosomal *fabI* gene from wild-type *E. coli* K12 in the pCC1FOS fosmid vector to prevent ENR overexpression effect. This experiment was performed in triplicate for each antibiotic concentration.

### General DNA manipulations

Standard recombinant DNA techniques were followed as previously described^34^. DNA sequencing and primer synthesis were performed commercially at the DNA sequencing facility of MacroGen (Seoul, Korea). Sequence comparisons (nucleotides/amino acids) were performed using the BLAST and ORF finder online services provided by the National Center for Biotechnology Information (NCBI http://blast.ncbi.nlm.nih.gov). Multiple alignment analysis was performed using BioEdit software in combination with GeneDoc.

### Metagenomic library construction and screening for TCS-resistant clones

The metagenomic library from alluvial soil (AS) was previously constructed^35^,^36^; however to construct the metagenomic library from the industrially contaminated area, soil samples were collected from the Gam-geon stream (Sasang-Gu, Busan, Republic of Korea), which receives combined sewer effluent from various industries, as this area has been highly urbanized by a number of industries since 1968. The Gam-geon stream finally meets the Nakdong River, which in turn converges to the East Sea, a marginal sea of the Pacific Ocean, and forms a unique ecosystem. Soil DNA was isolated as previously described^37^. Both of the soil sample collection sites for metagenomic library construction were presumed to be TCS contaminated due to anthropogenic processes. The metagenomic library was constructed and stored following the protocol described previously^35^.

To select TCS-resistant clones from the fosmid library, the library pool stocks were diluted in a buffer (per liter: NaCl, 8.5 g; KH_2_PO_4_, 0.3 g; Na_2_HPO_4_, 0.6 g; MgSO_4_, 0.2 g; gelatin, 0.1 g), and fosmid clones from the metagenomic library were spread on LB agar containing 30 μg/ml chloramphenicol and 5 μg/ml TCS. *E. coli* colonies that grew on LB with TCS were picked and further tested at higher concentrations of TCS until they were unable to grow or were found refractory to TCS. Pure cultures of TCS-resistant clones were processed for fosmid isolation followed by BamHI restriction digestion, and the unique clones were finally selected based on BamHI restriction profiles.

### Subcloning of TCS-resistant clones

Secondary library was constructed in pUC119 following the previously described procedure^38^ and TCS resistant subclones (primary subclones) were selected. Once the nucleotide sequence analysis of the TCS-resistant clone was completed, the candidate gene(s) for TCS resistance were further subcloned into pUC119 or the pGEM®-T Easy vector (secondary subclones) and tested at a similar concentration of TCS as the parental clones. Metagenomic TCS-resistant clones that failed to produce subclones with restriction digestion were processed using Tn-5 transposon mutagenesis using the EZ-Tn5™ <KAN-2> Insertion Kit (Epicentre) to select for the genes responsible for TCS resistance. Transposon mutagenesis was performed according to the manufacturer’s protocol. Mutants that were unable to grow on TCS-containing medium were selected, and transposon insertion sites were sequenced.

### Complementation analysis

To confirm the ENR activity of various metagenome-derived ENR homologues and other candidate clones, complementation studies were performed. Recombinant plasmids carrying candidate ENR homologue genes from metagenomic clones were transferred to a temperature-sensitive *fabI* mutant of *E. coli* JP1111^39^. This mutant has a mutation in FabI ENR that renders it unable to grow at the non-permissive temperature (42 °C). *E. coli* JP1111 containing the fosmid or metagenomics clones were grown in triplicate on LB agar medium with IPTG, and bacterial growth was observed at 30 °C and 42 °C. Bacterial growth of *E. coli* JP1111 at 42 °C for 48 h indicated complementation of FabI ENR activity.

To confirm the 3-oxoacyl-acyl-carrier-protein reductase (FabG) activity of two of the metagenomic clones, complementation studies were performed in a similar manner as described for ENR complementation but using a *fabG* temperature-sensitive mutant of *E. coli* CL37^40^. This mutant has two mutations (A154T and E233K) in its FabG reductase, rendering it unable to grow at the non-permissive temperature (42 °C).

### Co/Cross- and multiple-resistance tests

To investigate whether *E. coli* DH5α with various metagenomic clones carrying TCS resistance showed any differential responses to a variety of antibiotics, we first grew these cells to an OD_600_ of 1.0 in the presence or absence of 0.1 μg/ml TCS, a sub-lethal concentration (to observe cross-resistance). These bacterial cultures were then serially diluted to 1 × 10^5^ CFU/ml, and 3 μl of each cell suspension (1 × 10^5^ CFU/ml) was spotted onto LB agar medium containing the antibiotics to be tested for co/cross-resistance. LB plates were incubated at 37 °C for three days, and bacterial colony formation was examined at regular intervals of 24 h. The lowest concentration of antibiotic that prevented bacterial growth was considered the MIC for that antibiotic. This experiment was performed in triplicate for each concentration of each antibiotic. Prior to testing for co/cross-resistance, two independent experiments were performed in which a set of TCS-resistant clones was cultured in LB broth supplemented with 0.1 μg/ml TCS (for cross-resistance studies) with an identical set not exposed to TCS (to observe co-resistance). This experiment was performed in triplicate for each concentration of each antibiotic. MIC of *E. coli* carrying TCS resistance genes was determined to 19 different antibiotics of 8 different classes (Supplementary Table S19).

### Phylogenetic and taxonomic analysis

Phylogenetic analysis was performed as described previously^41^ for unique metagenomic ENR groups using amino acid sequences of metagenomic ENRs, previously known, well-characterized ENRs and other protein homologues that shared maximum identity to specific metagenomic ENR types. Sequence similarity searches were performed using the UniRef50 database for the well-characterized ENRs (FabL^42^, FabI^43^, and protein homologues other than ENRs, including FabG^40^ and 7-α-hydroxysteroid dehydrogenase (7-α-HSDH)^44^ from *E. coli* and *Comamonas testosteroni*^45^ and for the unique metagenomic ENR candidate groups. This analysis resulted in sequences that were more than 50% identical. For each homology search, the top 10 scoring entries were selected. All identified sequences were compiled together with closely identical corresponding metagenomic ENRs, and redundant sequences were removed using the online Decrease Redundancy program^46^. The sequences were then aligned with MEGA 6^47^ using the MUSCLE algorithm^48^. The alignment output was analyzed using the maximum likelihood method in MEGA, utilizing the nearest-neighbor-interchange strategy, which allows for deletion of gaps that exist in less than 50% of the sequences, and 500 bootstrap replicates to evaluate the confidence. The taxonomic origin of functionally selected DNA fragments was determined using RAIphy, a composition-based classifier that can accurately predict taxonomy without a strict reliance on phylogenetically close sequences in public databases compared to other similarity-based methods^49^. To predict the source phylum of resistance-conferring soil DNA fragments, we used all of the assembled metagenomic fragments, seeded predictions using the RAIphy 2012 RefSeq database, and binned DNA fragments with the ‘iterative refinement’ option in RAIphy.

### Selection of environmental metagenomic datasets

To investigate the abundance and diversity of genes encoding ENR homologues in presumably TCS-contaminated and TCS-free environments, 49 metagenome datasets were selected from 13 different environments. The presumably TCS-rich environments (TCSREs) included 3 samples from WWTPs (from an activated sludge WWTP in China, a tannery WWTP in China, and a WWTP in Hong Kong) and one sample from the ocean sediment in China. The presumably TCS-free or low-TCS environments included 9 samples: 2 human oral samples (ancient human oral sample from an archeological site and a human oral sample from Spain), 4 fresh water samples (from Antarctica, Minnesota, Australia and South Australia), 2 forest soil samples (a Mediterranean forest soil from Spain and a Temperate forest soil from Finland) and one glacier sample (from Austria). The metagenomic data sets for these samples were downloaded from the public repository MG-RAST web site (http://metagenomics.anl.gov/). Detailed information about how metagenomic data sets were selected and about the TRG reference database can be found in Supplementary Data 2.

### Comparative search for ENR diversity and abundance in environmental metagenomic datasets using the TRG reference database

A TRG reference database was constructed. The TRG database contained the deduced amino acid sequences of well-known prototypic and metagenome-derived ENRs identified in this study (Supplementary Data 2). TRG sequence reads were identified by performing a homology search between the environmental metagenomic datasets (query as nucleotide sequences) and the TRG reference database (subject as protein sequences) using BLASTx. Annotated sequence reads were removed if their e-value was lower than 10e^−10^. The annotated read numbers (hits) from individual metagenomic datasets were normalized as previously described^5^. Briefly, to normalize the annotated read number (hits) per metagenome, we considered the average read length, the TRG reference sequence length, the 16S rRNA gene sequence length and the 16S rRNA sequence hits identified from metagenomic datasets in the Greengenes database (retrieved from MG-RAST)^5^. The TRG normalized abundance data are described in Supplementary material Fig. 5.xlsx.

Normalization of the data for each dataset was performed as described using the following equation^5^:


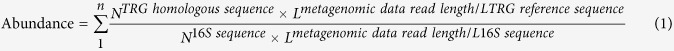


where *N*^*TRG homologous sequence*^ is the number of annotated TRG-like sequences, annotated as one specific TRG reference sequence; *L*^*TRG reference sequence*^ is the sequence length of the corresponding specific ENR reference sequence in the TRG database; *N*^*16S sequence*^ is the number of 16S rRNA gene sequences identified in the metagenomic data; *L*^*16S sequence*^ is the average length of the 16S rRNA gene sequence found in the Greengenes database (1,432 bp); *n* is the number of mapped TRG reference sequences; and *L*^*metagenomic data read length*^ is the sequence length for the corresponding sequence technology used, such as Illumina HiSeq, Sanger sequencing, or 454 pyrosequencing (refer to Supplementary Data 2).

### Analysis of normalized abundance of ENR homologues from environmental metagenomes

All comparisons of the relative abundance of TRG homologues between metagenomes were performed using the normalized abundance reads for each ENR divided by the total number of normalized ENR reads in the dataset (Supplementary material Fig. 5.xlsx). To compare the dispersion of TCS resistant ENRs in various environments (metagenomes), principal coordination analysis (PCoA) was performed using the Bray-Curtis dissimilarity measures for the TRG subtypes. All statistical analyses were performed with R software (version 3.2.2) (http://www.r-project.org/) using Vegan^50^ and ggplot2^51^ packages (Supplementary material Fig. 5.xlsx, Supplementary material Fig. 5d.xlsx).

## Additional Information

**How to cite this article**: Khan, R. *et al*. Triclosan Resistome from Metagenome Reveals Diverse Enoyl Acyl Carrier Protein Reductases and Selective Enrichment of Triclosan Resistance Genes. *Sci. Rep.* **6**, 32322; doi: 10.1038/srep32322 (2016).

## Supplementary Material

Supplementary Information

Supplementary Data 1

Supplementary Data 2

Supplementary material Figure 5

Supplementary material Figure 5D

## Figures and Tables

**Table 1:** List of metagenomic TCS resistant clones/subclones from alluvial soil and industrially contaminated soil.

No.	OMC	PSC	SSC	Source	Determinant for TCS^R^	ID^£^ (%)	Remarks	MIC (μg/ml)	CMP
1	pAT4	pAT4-2	pAT4-3	AS	YX6K-type FabI	42	Prototypic FabI homologue	10	+
2	pAY2	pAY2-1	pAY2-2	AS	YX6K-type FabI	99	Prototypic FabI homologue	350	+
3	pAL1	pAL1-4	pAL1-5	ICS	YX6K-type FabI	30	Prototypic FabI homologue	15	+
4	pX	pX-1	pX-1N	ICS	YX6K-type FabI	33	Prototypic FabI homologue	65	+
5	pAZ2	pAZ2-1	—	AS	YX6K-type FabI	50	Prototypic FabI homologue	75	+
6	pAB1	pAB1-2	pAB1-3	ICS	YX7K-type ENR	25	Novel candidate ENR	30	+
7	pAF1	pAF1-5	pAF1-6	ICS	YX7K-type ENR	27	Novel candidate ENR	20	+
8	pBF1	pBF1-4	pBF1-5	AS	7-α-HSDH homologue	41	Novel candidate	650	+
9	pL	pL-1	pL-2	ICS	7-α-HSDH homologue	41	Novel candidate	650	+
10	pY	pY-3	pY-4	ICS	7-α-HSDH homologue	41	Novel candidate	650	+
11	pR-2	pR-2-2	pR-2-3	AS	7-α-HSDH homologue	40	Novel candidate	650	+
12	pQ-2	pQ-2-2	pQ-2-3	AS	7-α-HSDH homologue	41	Novel candidate	650	+
13	pQ2N	pQ2N-1	pQ2N-2	AS	7-α-HSDH homologue	41	Novel candidate	650	+
14	pBC1	pBC1-1	—	AS	7-α-HSDH homologue	41	Novel candidate	650	+
15	pD-1	pD-1-6	pD-1-7	AS	FabG homologue	31	Novel candidate	650	+
16	pAM1	pAM1-3	pAM1-4	AS	FabG homologue	29	Novel candidate	650	+
17	pA	pA-2	pA-3	AS	YX8K-type FabV	52	Prototypic FabV homologue	650	+
18	pAQ2	pAQ2-2	pAQ2-3	AS	YX8K-type FabV	63	Low resistance and compromised ENR activity	15	—
19	pJ	pJ-1-4	pJ-1-5	AS	YX8K-type FabV	74	Prototypic FabV homologue	650	+
20	pB	pB-1-4	pB-1-5	AS	YX8K-type FabV	57	Prototypic FabV homologue	650	+
21	pAV2	pAV2-2	pAV2-3	AS	YX6K-type FabL	32	Prototypic FabL homologue	50	+
22	pAU2	pAU2-3	pAU2-4	AS	YX6K-type FabL	31	Prototypic FabL homologue	40	+
23	pAY4-2	pAY4-3	—	AS	FabK ENR homologue	27	Low resistance and compromised ENR activity	20	—
24	pAH4	pAH4-3	pAH4-4	ICS	Novel hypothetical protein like ENR candidate	—	Novel candidate	20	+
25	pBB2	pBB2-1	—	ICS	*acrB* gene homologue	52	Novel candidate	15	—
26	pW	pW-1	—	ICS	Unknown	—	—	20	—
27	pAX1	—	—	AS	Unknown	—	—	5	—
28	pAY4-1	—	—	AS	Unknown	—	—	20	—
29	pBE3	—	—	ICS	Unknown	—	—	10	—
30	pAP3	—	—	ICS	Unknown	—	—	10	—
31	pAW1	—	—	ICS	Unknown	—	—	10	—
32	pAG2	—	—	AS	Unknown	—	—	40	-

Symbols and Abbreviations: OMC, original metagenomic clone; PSC, Primary subclone; SSC, Secondary subclone; TCSr, Triclosan resistance; ICS, Industrially contaminated soil; AS, Alluvial soil; £, Identity with the closest candidate ENR type/functional protein; ENR, Enoyl ACP Reductase; 7-α-HSDH, 7-α-hydroxysteroid dehydrogenase homologue; CMP, complementation of the ENR activity.
