# Supplementary material for: Triclosan Resistome from Metagenome Reveals Diverse Enoyl Acyl Carrier Protein Reductases and Selective Enrichment of Triclosan Resistance Genes
Source: Sci Rep. 2016 Aug 31;6:32322. doi: 10.1038/srep32322 (PMC5006077; doi:10.1038/srep32322)
Supplement: Supplementary Information [file srep32322-s1.pdf]

Triclosan Resistome from Metagenome Reveals Diverse Enoyl Acyl Carrier Protein Reductases and Selective Enrichment of Triclosan Resistance Genes

Running title: Triclosan resistome from the metagenome

Raees Khan[1†], Hyun Gi Kong[1 †], Yong-Hoon Jung[2], Jinhee Choi[2], Kwang-Yeol Baek[2], Eul Chul Hwang[1] and Seon-Woo Lee[1.2]*

[1]*Department of Applied Biology, Dong-A University, Busan 604-714, Republic of Korea*

[2]*Department of Applied Bioscience, Dong-A University, Busan 604-714, Republic of Korea*

[†]These authors contributed equally to this work.

*Corresponding author

Seon-Woo Lee, Ph.D
Professor
Department of Applied Bioscience
Dong-A University
Busan 49315
Republic of Korea
Email: seonlee@dau.ac.kr
TEL: +82-51-200-7551
FAX: +82-51-200-7505

Guide for supplementary information of the manuscript

1. Supplementary Information

This file contains Supplementary Figures and Supplementary Tables combined. Supplementary figures are placed first followed by Supplementary tables. There are total 7 Supplementary figures, labelled as Supplementary Figure S1-Supplementary figure S7. Supplementary figures are followed by 31 Supplementary Tables, labelled as Supplementary Table S1-Supplementary Table S31.

Other files provided as a single supplementary data files include

2) Supplementary Data 1.xlsx

This file consists of two spread sheets. The first spreadsheet contains gene information indicating the presence of metagenome derived substitutions in FabI ENRs from 372 well-known human-associated pathogenic and nonpathogenic bacterial strains. The second spread sheet contains same information for 29 well known soil borne plant pathogenic strains. Additional necessary information about individual strains is also mentioned.

3) Supplementary Data 2.xlsx

This file consists of two spread sheets. The first spreadsheet has detailed information about the metagenomic data sets retrieved from public repository site, MG-RAST, such as nature and origin of the sample, sequencing method used, project etc. The second spreadsheet represents information about TRG-Reference database, such as information about the prototypic and metagenomic ENRs used for constructing this database.

4) Supplementary material Figure 5.xlsx

This file consists of 12 spread sheets, enclosing information on BLASTx search result of ENR homologues from metagenome dataset, normalized data from each corresponding metagenomic environment, normalized abundance data, and ENR abundance data enclosed for Figure 5-a,b,c,e,f.

5) Supplementary materials Figure 5D.xlsx

This file contains the R script information, mapping file and additional data using which Principal coordinate analysis (PCoA) plot was made for Figure 5-d.

Supplementary Figures and Supplementary Tables

[Supplementary Figure. S1]

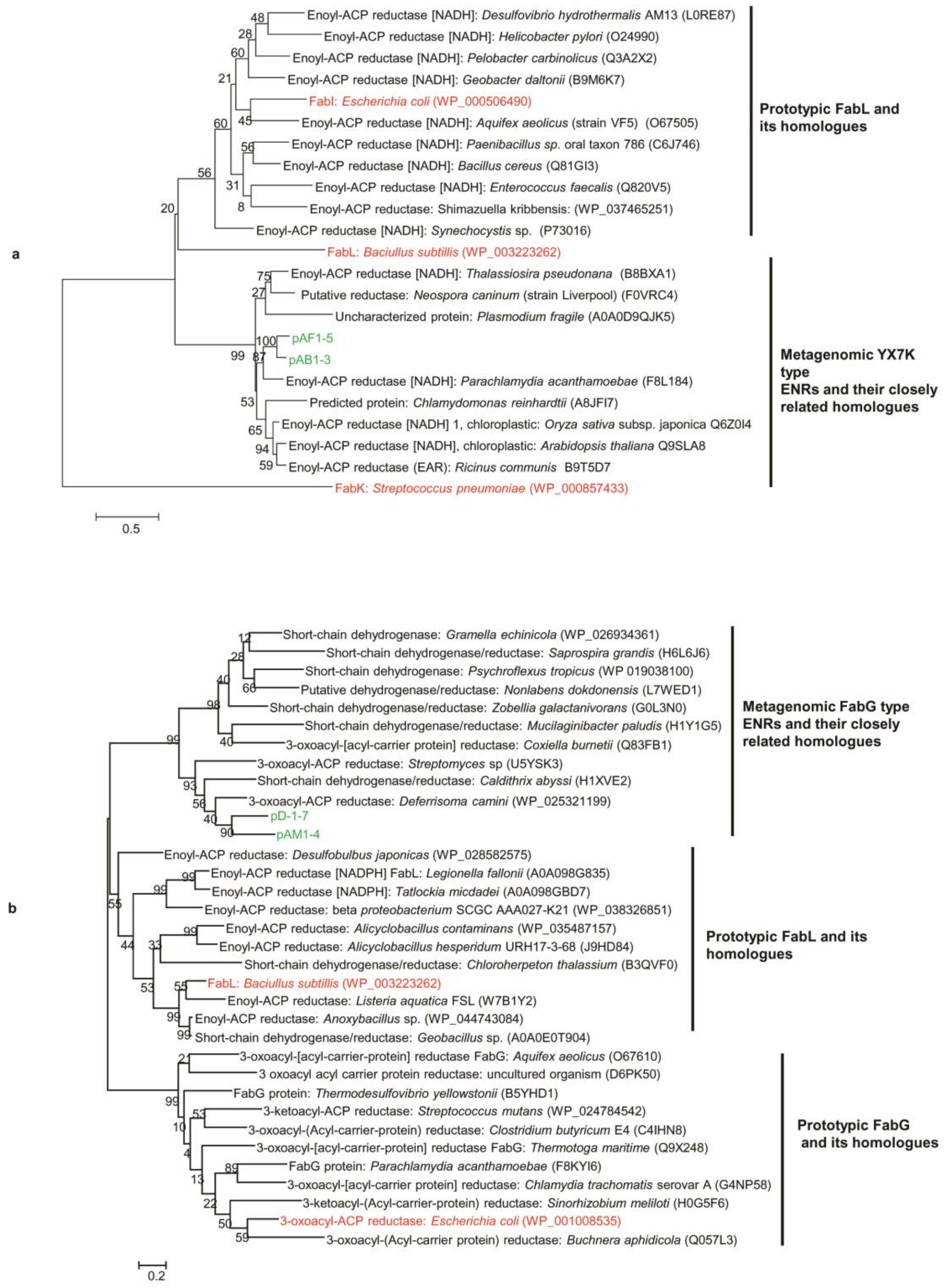

Supplementary Figure S1. The metagenomic YX7K-type ENRs and 3-oxoacyl-ACP reductase (FabG)-like ENRs and their homologues clustered as a separate clade from other closely related prototypic ENRs. a, Phylogenetic tree of metagenomic YX7K-type ENRs; pAF1-5 and pAB1-3 (green), FabI, FabL homologues and FabK as an outgroup. Maximum likelihood analysis of the well-characterized FabI, FabL and FabK enzymes (red) and homologues of metagenomic YX7K-type ENRs and closely related FabI ENRs with sequence identity over 50% from the Uniref50 database. Bootstrap values are indicated for each node in a bootstrap analysis of 500 replicates. The scale bar represents 0.5 estimated amino acid substitutions per residue. **b**, Phylogenetic tree of metagenomic 3-oxoacyl-ACP reductase (FabG) type ENRs (green), prototypic 3-oxoacyl-ACP reductase and closely related FabL. Maximum likelihood analysis of well-characterized 3-oxoacyl-ACP reductase and FabL enzymes (red) and homologues of metagenomic 3-oxoacyl-ACP reductase-type ENRs and prototypic FabL ENRs with sequence identity over 50% from the Uniref50 database. Bootstrap values are indicated for each node in a bootstrap analysis of 500 replicates. The scale bar represents 0.2 estimated amino acid substitutions per residue.

[Supplementary Figure. S2]

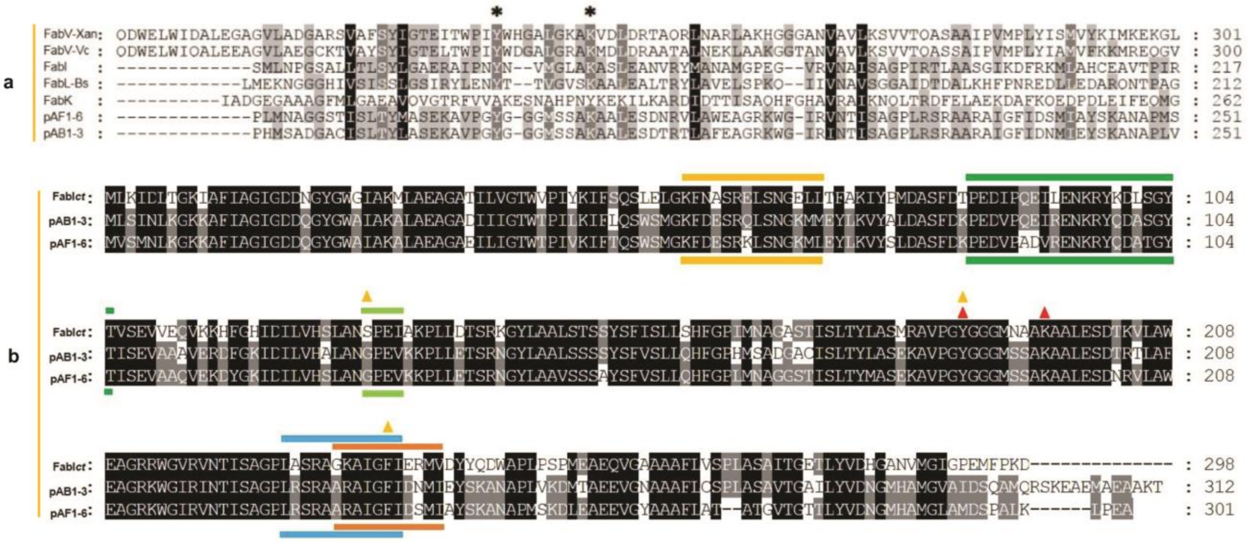

Supplementary Figure S2. Metagenomic triclosan-resistant ENRs with a YX7K-type catalytic domain. a, Multiple alignment (partial) of metagenomic YX7K-type ENRs with known prototypic bacterial ENRs. The known prototypic ENRs include the FabV ENRs from *Xanthomonas oryzae* (FabV-Xan) and *Vibrio cholerae* (FabV-Vc), FabI from *E. coli* (FabI), FabL from *Bacillus subtilis* (FabL-Bs), and FabK from *Streptococcus pneumoniae* (FabK). Metagenomic YX7K-type ENRs shared 25-30% maximum identity with *E. coli* FabI and *B. subtilis* FabL ENRs. The active site tyrosine and lysine residues are indicated with asterisks. Unlike other prototypic ENRs, the metagenome-derived ENR homologues carried a different YX7K-type catalytic domain. **b**, Multiple alignment of metagenomic YX7K-type ENRs with *Chlamydia trachomatis* ENR (on top). The YX7K-type active site tyrosine and lysine residues are indicated with red triangles. Two additional loops that are not present in closely related prototypic FabIs are indicated by orange and dark green bars. The flipping loop, the substrate binding loop, and the lid loops are indicated in cyan, sandy brown and lime green bars,

respectively. Residues Y_{187}, S_{129} and F_{234} of FabI*ct* interact with antibacterial AFN1252 and are indicated by orange pyramids.

[Supplementary Figure. S3]

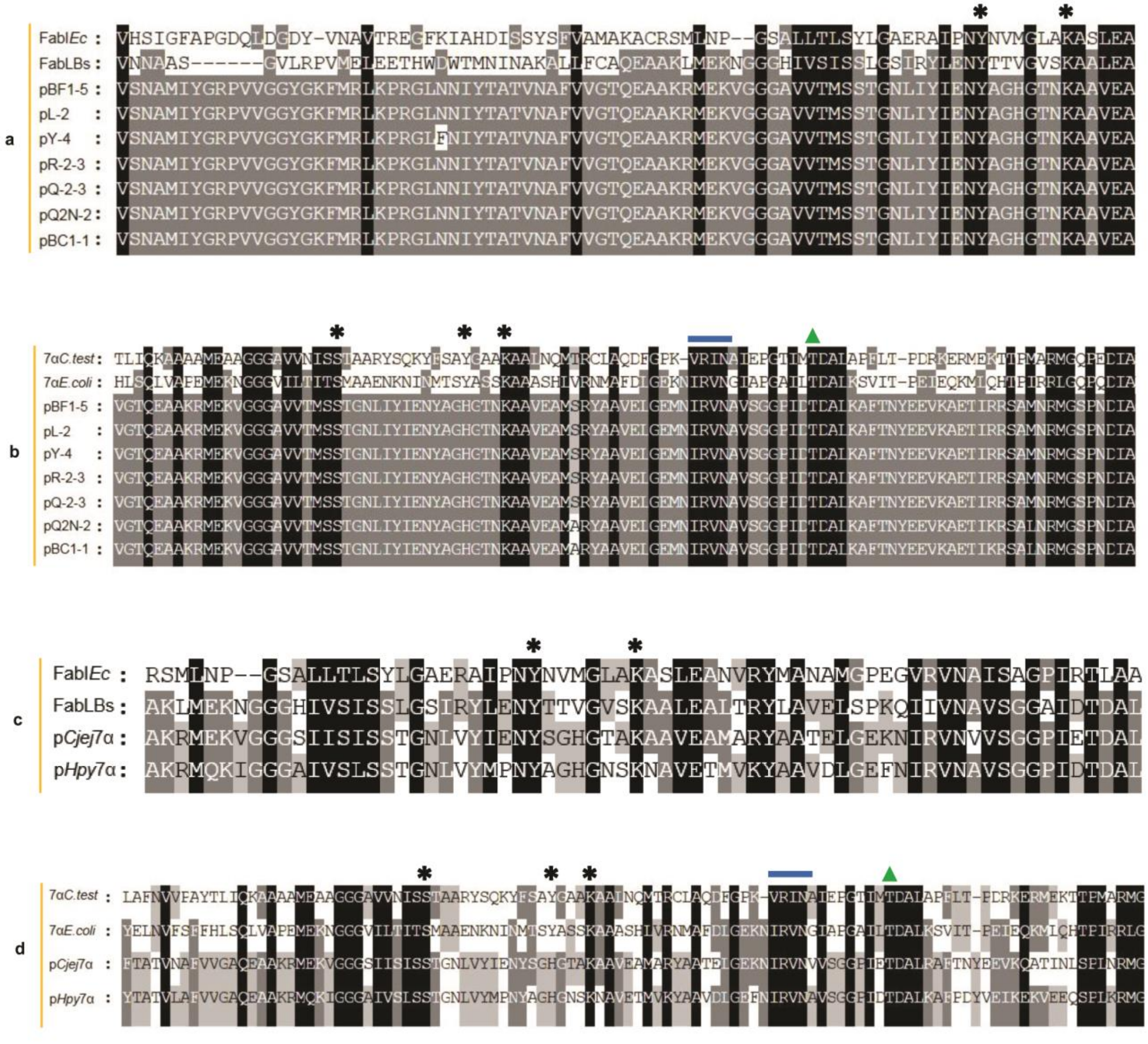

Supplementary Figure S3. Metagenomic 7-α-HSDH-like ENR homologues, which are present in Epsilonproteobacteria, share similar structural features with closely related well-characterized 7-α-HSDH and with prototypic FabI and FabL ENRs. a, Multiple

alignment (partial) of metagenomic 7-α-HSDH-like ENR homologues with *E. coli* FabI and *B. subtilis* FabL. The metagenomic 7-α-HSDH homologues shared low similarities with FabI and FabL but still shared a similar catalytic domain. The active site tyrosine and lysine residues are indicated with asterisks. **b**, Multiple alignment (partial) of metagenomic 7-α-HSDH-like ENRs with 7-α-HSDH from *C. testosteroni* and *E. coli* (on top). The residues involved in the catalytic function of the enzyme are indicated with asterisks. In metagenomic 7-α-HSDH-like ENRs, the Y_{159}/Y_{161} residue (in *E. coli* and *C. testosteroni,* respectively), which acts as a basic catalyst, has been replaced with a histidine, whereas the other two residues, S_{146}/S_{148} and K_{163}/K_{165}, are strictly conserved among these 7-α-HSDH-like metagenomic ENRs. The RINA-like sequences, necessary for reaction direction, are indicated by blue bars, whereas the conserved threonine is indicated by a green triangle. **c,** Multiple alignment (partial) of prototypic FabI from *E. coli* and FabL from *B. subtilis* (on top) with 7-α-HSDH from *C. jejuni* and *H. pylori*. The active site tyrosine and lysine residues are indicated with asterisks. **d**, Multiple alignment (partial) of 7-α-HSDH-like ENRs from *C. jejuni* and *H. pylori* with *E. coli* and *C. testosteroni* 7-α-HSDH (on top). The residues involved in the catalytic function of the enzyme are indicated with asterisks. Similar to metagenomic 7-α-HSDH-like ENRs, the Y_{159}/Y_{161} residue (of *E. coli* and *C. testosteroni*, respectively), which acts as a basic catalyst, has been replaced by histidine, whereas the other two residues, S_{146}/S_{148} and K_{163}/K_{165}, are strictly conserved among these 7-α-HSDH-like ENRs. The RINA-like sequence, necessary for reaction direction, is indicated by a blue bar, whereas the conserved threonine is indicated by a green triangle.

[Supplementary Figure. S4]

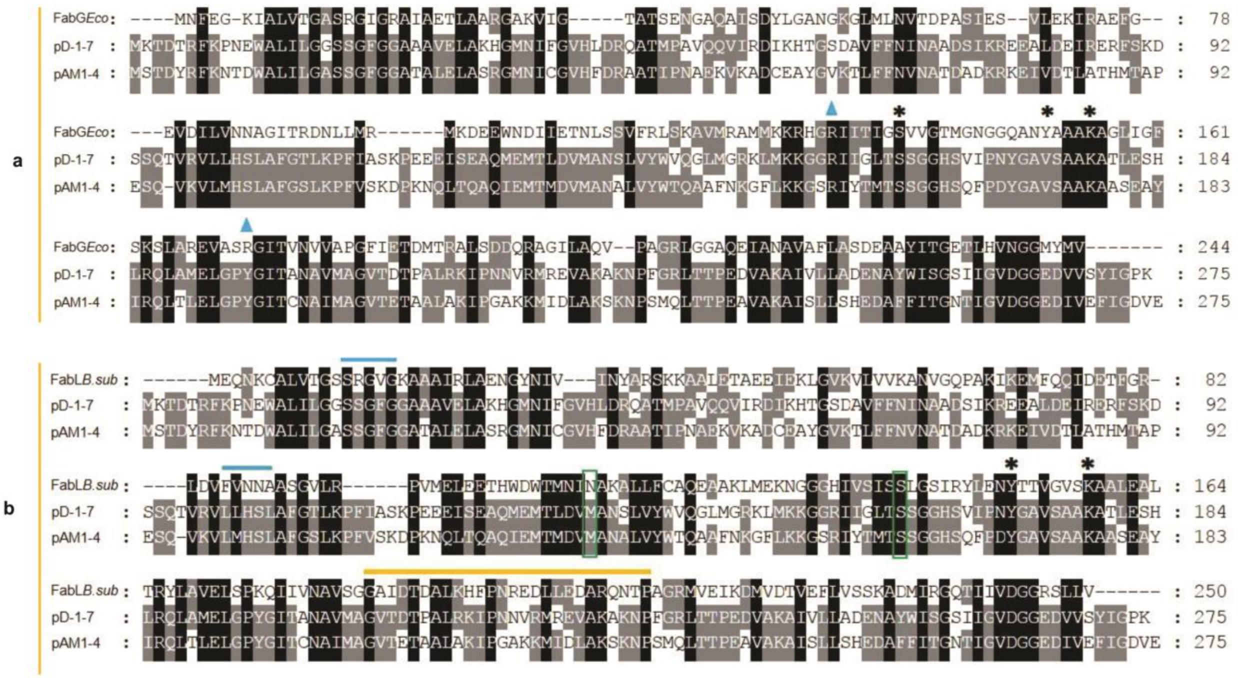

Supplementary Figure S4. Metagenomic 3-oxoacyl-[acyl-carrier-protein] reductase (FabG)-like ENR candidates share common features with both the prototypic FabG of *E. coli* and the FabL of *B. subtilis*. a, Multiple alignment of FabG of *E. coli* (on top) with metagenomic FabG-like ENR candidates. The active site triad residues Ser_{138}, Tyr_{151} and Lys_{155} are indicated with asterisks, and Tyr_{151} is replaced with a nonpolar valine in the metagenomic FabG-like ENR candidates. Cyan triangles indicate the two arginine residues that are central to ACP-binding. **b**, Multiple alignment of the FabL ENR of *B. subtilis* with metagenomic FabG-like ENR candidates. The YX6K active site residues are indicated with asterisks. The substrate-binding region and the residues involved in cofactor binding are indicated by orange and cyan bars, respectively. The highly conserved Asn_{113} and Ser_{141} residues are boxed in green.

[Supplementary Figure. S5]

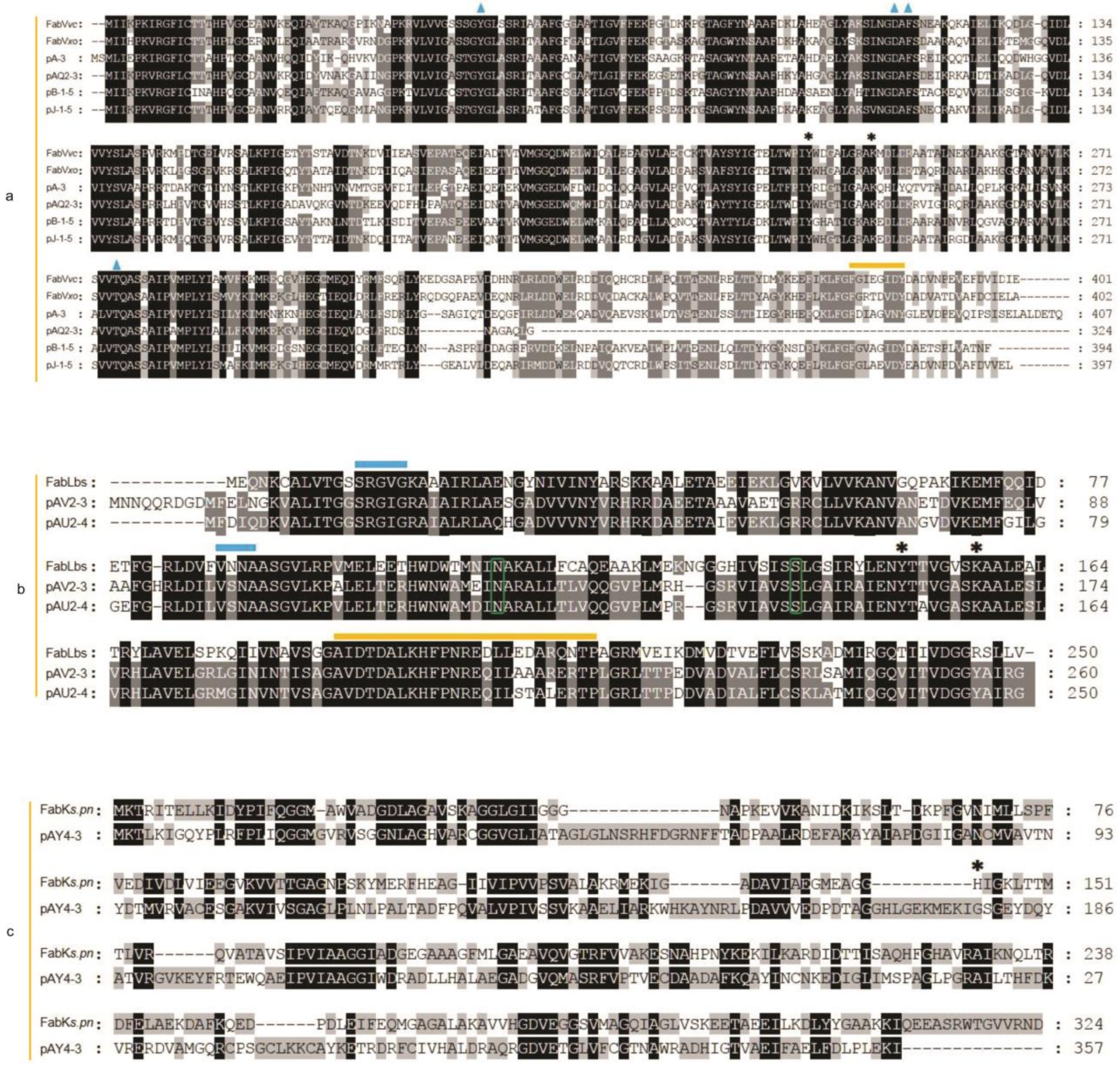

Supplementary Figure S5. Metagenomic FabV, FabL and FabK-like ENR homologues showed compromised ENR activity and lower resistance despite having shared structural similarities with previously known prototypic ENRs. a, Multiple alignment of metagenomic FabV ENR homologues with FabV from *V. cholerae* (FabV*vc*) and *X. oryza*

(FabV*xo*). The YX8K-type active site tyrosine and lysine residues are indicated with asterisks. Residues with key enzyme functions are indicated by cyan triangles. The conserved FAD-binding domain is indicated with an orange bar. **b,** Multiple alignment of metagenomic FabL ENR homologues with FabL from *B. subtilis* (on top). The YX6K-type active site residues are indicated with asterisks. The substrate-binding region is indicated by orange bars. Residues involved in cofactor binding are indicated by cyan bars. Highly conserved asparagine and serine residues are boxed in green. **c**, Multiple alignment of the metagenomic FabK homologue with FabK of *S. pneumoniae*. The conserved $histidine_{144}$ residue is indicated with an asterisk. This residue has been replaced by glycine in the metagenomic FabK-like ENR homologue.

[Supplementary Figure. S6]

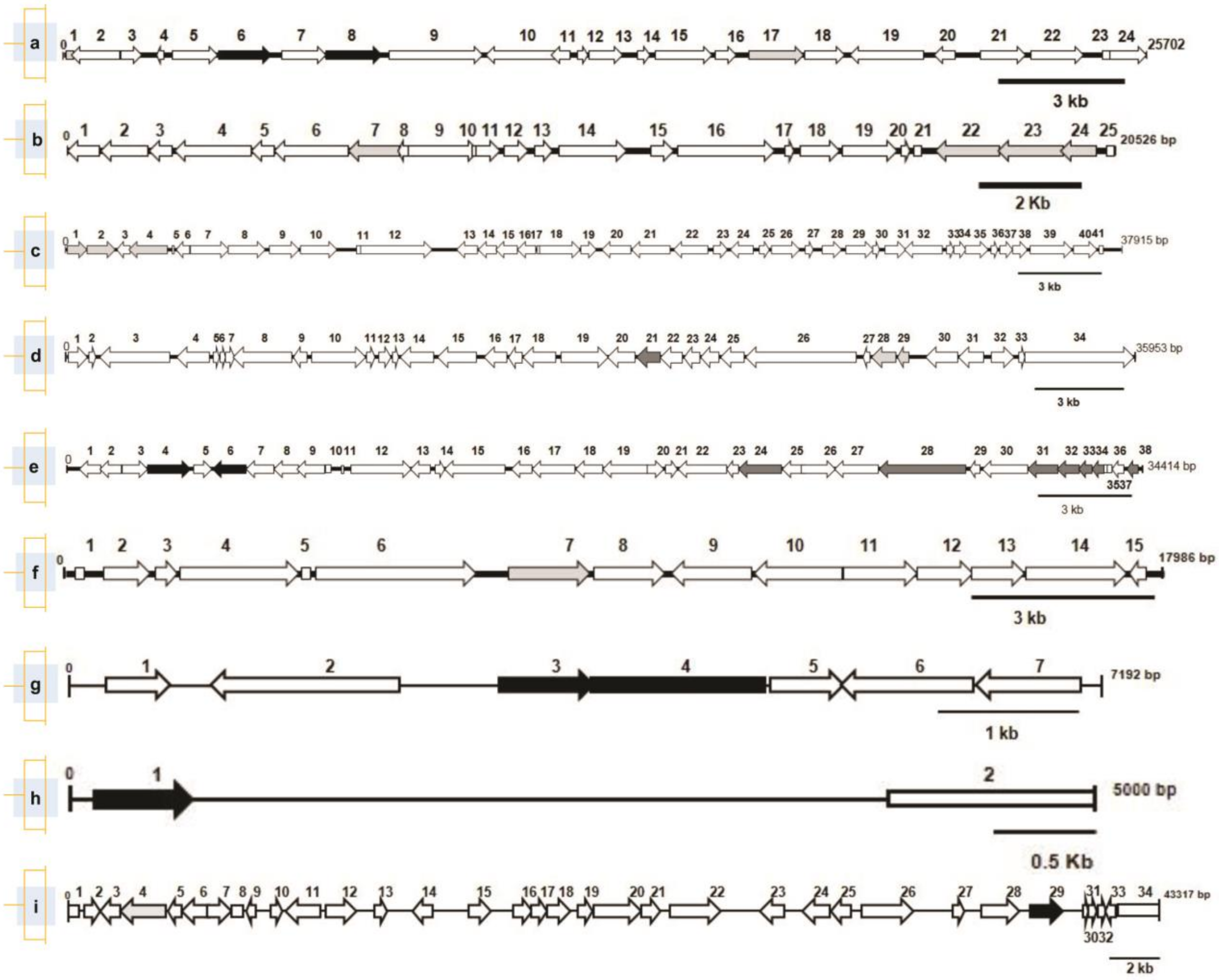

Supplementary Figure S6. Non-ENR genes conferred resistance to TCS in some metagenomic clones. In each ORF map, a DNA size scale is shown at the bottom right. All of the black- or gray-marked ORFs are described below. **a**, Genetic organization of ORFs in clone pBE3. Putative ORFs are listed from 1 to 24: $orf_{6,8}$ encodes MFS transporter homologues, whereas $orf_{1,17}$ encodes a mobile element signature homologue. **b**, Genetic organization of ORFs in clone pAY4-1. Putative ORFs are listed from 1 to 25, and $orf_{7,22\text{-}24}$ encode efflux transporter protein homologues. **c**, Genetic organization of ORFs in clone pAW1. Putative

ORFs are listed from 1 to 41, and *orf*$_{1,2,4}$ encode oxidases and photolyases, respectively. **d**, Genetic organization of ORFs in clone pAX1. Putative ORFs are listed from 1 to 34, and *orf*$_{21}$ encodes an alpha/beta hydrolase, whereas *orf*$_{28,29}$ encodes mobile element signatures. **e**, Genetic organization of ORFs in clone pAP3. Putative ORFs are listed from 1 to 38, and *orf*$_{4,6}$ encodes an MFS transporter and aldo-keto reductase homologues, respectively. The *orf*$_{24,28,31-34,38}$ encodes mobile element signatures. **f**, Genetic organization of ORFs in clone pAG2. Putative ORFs are listed from 1 to 15, and *orf*$_{7}$ encodes an MFS transporter homologue. **g**, Genetic organization of ORFs in metagenomic clone pW-1. Putative ORFs are listed from 1 to 7, and *orf*$_{3,4}$ encode ABC transporter efflux homologues. **h**, Genetic organization of ORFs in clone pBB2-1. Putative ORFs are listed from 1 to 2, and *orf*1 encodes a TCS-resistant AcrB efflux protein homologue that was 5 times smaller than prototypic AcrB proteins. **i**, Genetic organization of ORFs in clone pAH4. Putative ORFs are listed from 1 to 34, and *orf*$_{29}$ encodes a TCS-tolerant novel-hypothetical-protein-homologue-like ENR candidate. *orf*$_{4}$ encodes an ABC transporter that might be responsible for the observed co-resistance to other antibiotics.

[Supplementary Figure. S7]

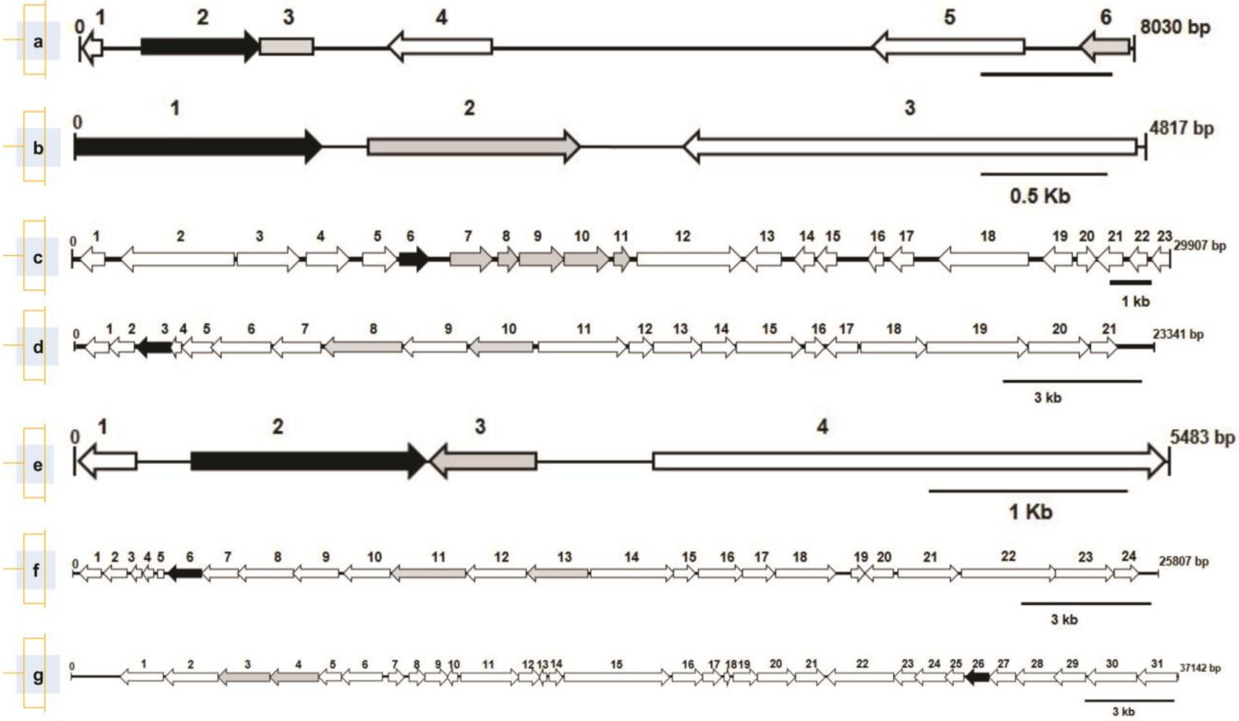

Supplementary Figure S7. Metagenomic TCS-resistant clones conferred co/cross-resistance to other antibiotics. In all ORF maps, a DNA size scale is shown at the bottom right**. a**, Genetic organization of ORFs in clone pAF1-5. Putative ORFs are listed from 1 to 6, and *orf*$_{2}$ encodes a YX7K-type ENR homologue. *orf*$_{3,6}$ encode a hydrolase and an exopolysaccharide synthesis protein that might be responsible for the observed co-resistance to other antibiotics. **b**, Genetic organization of ORFs in clone pAQ2-2. Putative ORFs are listed from 1 to 3, and *orf*$_{1}$ encodes a mildly TCS-tolerant FabV-type ENR homologue with compromised ENR activity. *orf*$_{2}$ encodes a beta-lactamase that may be responsible for the observed co-resistance to other antibiotics. **c**, Genetic organization of ORFs in clone pAV2. Putative ORFs are listed from 1 to 23, and *orf*$_{6}$ encodes a TCS-resistant FabL ENR homologue. *orf*$_{7,8,9,10,11}$ encode an ARG cluster, composed of different efflux pumps and antibiotic-

modifying enzymes that might be responsible for the observed resistance to other antibiotics. **d**, Genetic organization of ORFs in clone pL. Putative ORFs are listed from 1 to 21, and orf_3 encodes a 7-α-HSDH-like ENR homologue, whereas $orf_{8,10}$ encode ABC and MurJ efflux pump protein homologues, respectively. **e**, Genetic organization of ORFs in clone pB-1-4. Putative ORFs are listed from 1 to 4, and orf_2 encodes a TCS-resistant FabV ENR homologue, while orf_3 encodes a hydrolase. **f**, Genetic organization of ORFs in clone pBF1. Putative ORFs are listed from 1 to 24; orf_6 encodes a 7-α-HSDH-like ENR homologue, and $orf_{11,13}$ encode ABC and MurJ efflux pump protein homologues, respectively. **g**, Genetic organization of ORFs in clone pBC1. Putative ORFs are listed from 1 to 31, and orf_{26} encodes a 7-α-HSDH-like ENR homologue, while $orf_{3,4}$ encode AcrB efflux pump protein homologues. ORFs shown in black are genes encoding ENRs, and those in gray are candidate genes for co/cross-resistance to other antibiotics.

Supplementary Tables

Supplementary Table S1. Details of the source soil sample and metagenomic libraries constructed for TCS resistance screening.

Sample source	Sample code	No. of pools	No. of clones	pH	Organic (%)	Ca (mg/kg)	Mg (mg/kg)	K (mg/kg)	Na (mg/kg)	P (mg/kg)	Cu (mg/kg)
Industrially contaminated soil	ICS	87	78,600	6.58	14.98	2025.50	59.32	277.51	358.17	63.22	1055.00
Alluvial soil	AS	73	48,800	5.7	42.08	9661.05	9081.54	12218	982.15	1227.77	9.4

Supplementary Table S2. Taxonomic assignment of metagenome derived clone.

Clone Name	Assigned taxonomy by RAIphy
pA	***Proteobacteria***|*Gammaproteobacteria*|*Alteromonadales*|*Shewanellaceae*|*Shewanella*
pAB1	***Proteobacteria***|*Alphaproteobacteria*|*Rhodobacterales*|*Rhodobacteraceae*|*Pseudovibrio*
pAF1-5	***Proteobacteria***|*Gammaproteobacteria*|*Chromatiales*|*Chromatiaceae*|*Nitrosococcus*
pAH4	***Proteobacteria***|*Betaproteobacteria*|*Gallionellales*|*Gallionellaceae*|*Sideroxydans*
pAL1-4	***Proteobacteria***|*Alphaproteobacteria*|*Rhodobacterales*|*Rhodobacteraceae*|*Pseudovibrio*
pAM1	***Firmicutes***|*Bacilli*|*Bacillales*|*Bacillaceae*|*Bacillus*
pAQ2	***Proteobacteria***|*Betaproteobacteria*|*Burkholderiales*|*Burkholderiaceae*|*Cupriavidus*
pAT4	***Firmicutes***|*Bacilli*|*Bacillales*|*Alicyclobacillaceae*|*Sulfobacillus*
pAU2	***Proteobacteria***|*Gammaproteobacteria*|*Acidithiobacillales*|*Acidithiobacillaceae*|*Acidithiobacillus*
pAV2	***Proteobacteria***|*Gammaproteobacteria*|*Methylococcales*|*Methylococcaceae*|*Methylococcus*
pAY2-1	***Proteobacteria***|*Gammaproteobacteria*|*Enterobacteriales*|*Enterobacteriaceae*|*Escherichia*
pB-1	***Acidobacteria***|*Acidobacteria*|*Acidobacteriales*|*Acidobacteriaceae*|*Granulicella*
pBB2	***Chlamydiae***|*Chlamydiae*|*Chlamydiales*|*Simkaniaceae*|*Simkania*
pBF1-4	***Proteobacteria***|*Epsilonproteobacteria*|*Campylobacterales*|*Helicobacteraceae*|*Sulfuricurvum*
pD-1	***Verrucomicrobia***|*Opitutae*|*Puniceicoccales*|*Puniceicoccaceae*|*Coraliomargarita*
pJ	***Proteobacteria***|*Gammaproteobacteria*|*Aeromonadales*|*Aeromonadaceae*|*Aeromonas*
pL-1	***Proteobacteria***|*Epsilonproteobacteria*|*Campylobacterales*|*Helicobacteraceae*|*Sulfuricurvum*
pQ-2	***Proteobacteria***|*Epsilonproteobacteria*|*Campylobacterales*|*Helicobacteraceae*|*Sulfuricurvum*
pQ2N	***Proteobacteria***|*Epsilonproteobacteria*|*Campylobacterales*|*Helicobacteraceae*|*Sulfuricurvum*
pW-1	***Chlamydiae***|*Chlamydiae*|*Chlamydiales*|*Waddliaceae*|*Waddlia*
pX-1N	***Actinobacteria***|*Actinobacteria*|*Actinomycetales*|*Propionibacteriaceae*|*Microlunatus*
pY-3	***Proteobacteria***|*Epsilonproteobacteria*|*Campylobacterales*|*Helicobacteraceae*|*Sulfuricurvum*
pAZ2	***Proteobacteria***|*Deltaproteobacteria*|*Desulfuromonadales*|*Desulfuromonadaceae*|*Pelobacter*
pAY4-2	***Proteobacteria***|*Deltaproteobacteria*|*Desulfuromonadales*|*Geobacteraceae*|*Geobacter*
pBC1	***Proteobacteria***|*Epsilonproteobacteria*|*Campylobacterales*|*Helicobacteraceae*|*Sulfuricurvum*
pBE3-1	***unknown phylum***|*unknown class*|*unknown order*|*unknown family*|*Candidatus*
pAY4-1	***Proteobacteria***|*Deltaproteobacteria*|*Desulfuromonadales*|*Geobacteraceae*|*Geobacter*
pAW1	***Proteobacteria***|*Betaproteobacteria*|*Nitrosomonadales*|*Nitrosomonadaceae*|*Nitrosospira*
pAX1	***unknown phylum***|*unknown class*|*unknown order*|*unknown family*|*Candidatus*
pAP3	***Proteobacteria***|*Betaproteobacteria*|*Burkholderiales*|*Burkholderiaceae*|*Ralstonia*
pAG2	***Planctomycetes***|*Planctomycetacia*|*Planctomycetales*|*Planctomycetaceae*|*Planctomyces*
pR-2	***Proteobacteria***|*Epsilonproteobacteria*|*Campylobacterales*|*Helicobacteraceae*|*Sulfuricurvum*

Supplementary Table S3. Triclosan susceptibility based grouping of metagenome derived ENRs.

Groups	MIC (µg/ml)	Fold increase in MIC
Group I (388-722- fold increase in MIC) (Highly triclosan tolerant ENR homologues)		
pBC1-1*	650	722
pBF1-5*	650	722
pQ2N-2*	650	722
pQ-2-3*	650	722
pL-2*	650	722
pY-4*	650	722
pR-2-3*	650	722
pB-1-5*	650	722
pA-3*	650	722
pJ-1-5*	650	722
pAY2-2	350	388
pAM1-4*	650	722
pD-1-7*	650	722
Group II (22-83 fold increase in MIC) (Intermediate triclosan tolerant ENR homologues)		
pAZ2-1	75	83
pX-1N	65	72
pAB1-3	30	33
pAF1-6	20	22
pAU2-4	40	44
pAV2-3	50	55
pAY4-3	20	22
Group III (11-16 fold increase in MIC) (Mild triclosan resistant ENR homologues)		
pAQ2-3	15	16
pAT4-3	10	11
pAL1-5	15	16

Symbols; *, MIC was not determined for these clones, and the maximum level of triclosan, at which these clones were tested was 650 µg/ml. For the rest of the clones MIC was determined.

Supplementary Table S4. Presence of metagenome like YX7K type ENR homologues in obligate intracellular pathogenic bacteria.

**Organism**	**% ID**	**Pathogenicity**	**Host**	**ENR Accession Number**	**YX7K domain**
Waddlia chondrophila 2032/99	77	Human fetal death	Humans	CCB91849	+
Simkania negevensis	73	Pneumonia	Humans	WP_013943443	+
Candidatus Protochlamydia amoebophila	72	STD infections	Humans	CAF23876.1	+
Candidatus Protochlamydia amoebophila UWE25	72	No	Amoeba	YP_008151	+
Chlamydia psittaci	72	Psittacosis and RTI	Birds and humans	WP_020350159	+
Chlamydia pecorum	71	Reproductive disease	Koala and others	WP_013712757	+
Chlamydophila caviae	71	Eye infections	Guinea pigs	WP_011006351	+
Chlamydia psittaci 10_1398_11	71	Psittacosis and RTI	Birds and humans	EPP35064.1	+
Parachlamydia acanthamoebae	70	LRT	Humans	WP_013925330	+
Chlamydophila pneumoniae CWL029	70	CAP	Humans	NP_224606	+
Chlamydophila_pneumoniae_LPCoLN	70	RTI	Humans	ACZ33383.1	+
Chlamydophila_pneumoniae_AR39	70	RTI	Humans	AAF38201.1	+
Chlamydophila_pneumoniae_CWL029	70	RTI	Humans	NP_224606.1	+
Chlamydophila_pneumoniae_J138	70	RTI	Humans	BAA98614.1	+
Chlamydophila_pneumoniae_TW-183	70	RTI	Humans	AAP98351.1	+
Chlamydophila felis	70	Conjunctivitis	Cats and humans	WP_011458172	+
Chlamydia trachomatis L2c	69	STD infections	Humans	YP_004717235	+
Chlamydia trachomatis	69	STD infections	Humans	WP_009873559	+
Chlamydophila abortus S26/3	69	Abortion/fetal death	Mammals/humans	YP_219792	+
Chlamydia psittaci NJ1	69	Diarrhea	goats and sheep	YP_006740504	+
Chlamydia_trachomatis_434_Bu	69	STD infections	Humans	YP_001654443	+
Chlamydia suis	69	Conjunctivitis	Swine	WP_035405642	+
Chlamydia avium	69	Miscellaneous	Pigeons/birds	WP_038500200	+
Chlamydia_trachomatis_A_HAR-13	68	STD infections	Humans	AAX50356.1	+
Chlamydia_trachomatis_B_Jali20_OT	68	STD infections	Humans	CAX10551.1	+
Chlamydia_trachomatis_D_UW-3_CX	68	STD infections	Humans	NP_219607.1	+
Chlamydia_trachomatis_D-EC	68	STD infections	Humans	ADI50779.1	+
Chlamydia_trachomatis_D-LC	68	STD infections	Humans	ADI51791.1	+
Chlamydia_trachomatis_E_150	68	STD infections	Humans	ADH16869.1	+
Chlamydia_trachomatis_G_11222	68	STD infections	Humans	ADH18713.1	+
Chlamydia_trachomatis_E_11023	68	STD infections	Humans	ADH20562.1	+

Abbreviations and symbols; ID, Identity to metagenomic YX7K type ENRs; RTI, Respiratory tract infections; STD, Sexually transmitted disease; +, Present; LRT, Lower respiratory tract infections; CAP, Community-acquired pneumonia.

Supplementary Table S5. Presence of metagenome like YX7K type ENRs in apicomplexa obligate intracellular pathogens.

Organism	% ID	Disease	Host	ENR Accession Number	YX7K domain
Toxoplasma gondii	63	Toxoplasmosis	Humans/Animals	XP_002367432	+
Hammondia hammondi	63	Not reported	Warm blooded animals	KEP61065	+
Eimeria Tenella	59	Coccidiosis	Poultry	2PTG|A	+
Eimeria brunetti	54	Coccidiosis	Poultry	CDJ50922	+
Plasmodium cynomolgi strain B	52	Malaria	Primates	XP_004223347	+
Plasmodium knowlesi strain H	52	Malaria	Primates	XP_002261521	+
Plasmodium inui San Antonio	52	Malaria	Primates	EUD68469	+
Plasmodium chabaudi chabaudi	51	Malaria	Rodents	XP_745177	+
Plasmodium Berghei	51	Malaria	Mammals	3F4B_A	+
Neospora caninum Liverpool	50	Neosporosis	Canids and cattles	XP_003886297	+
Plasmodium Falciparum	46	Malaria	Humans	2O2Y_A	+

Abbreviations and symbols; ID, Identity to metagenomic YX7K type ENRs; +, Present.

Supplementary Table S6. Organisms with metagenomic 7-alpha-hydroxysteroid dehydrogenase (7-α-HSDH) like ENR homologues.

Organism	ENR Accession Number	% Identity to 7-α-HSDH	Importance of the organism	Potential host
Sulfuricurvum sp.	WP_015653931	96	Sulfur oxidinzing	None
Nitratifractor salsuginis	WP_013553872	82	Denitrifying bacteria	None
Arcobacter butzleri	WP_004509057	74	Potential enteropathogen	Humans/others
Arcobacter cibarius	WP_024776121	73	Potential enteropathogen	Broiler
Nautilia profundicola	WP_015902546	74	Sulfur-reducing bacteria	None
Arcobacter nitrofigilis	WP_013135927	70	Symbiotic organism	None
Campylobacter showae	WP_002950625	72	Oral cavity gingivitis	Humans
Campylobacter rectus	WP_004320005	71	Chronic periodontitis	Humans
Campylobacter concisus	WP_021089130	71	Emergent pathogen	Humans
Caminibacter mediatlanticus	WP_007474398	72	Chemolithoautotroph	None
Campylobacter upsaliensis	WP_004277624	70	Gastroenteritis and bacteremia	Pets/humans
Sulfurospirillum arcachonense	WP_024954653	69	Sulfur reduction	None
Campylobacter jejuni	WP_004297961	69	Food borne pathogen	Humans
Sulfuricurvum kujiense	WP_013459792	70	Sulfur-oxidizing bacterium	None
Sulfurospirillum barnesii	WP_014769378	70	Arsenate-reducing	None
Campylobacter curvus	WP_018136240	70	Bloody or chronic diarrhea.	Humans
Helicobacter pylori	WP_025275758	69	Cause Gastric Ulcer	Humans
Helicobacter Magdeburgensis	KGL14935	70	Potential intestinal pathogen	Mice
Helicobacter pametensis	WP_027327035	67	Found in birds and swine	Not known
Helicobacter macacae	WP_023926757	68	Colitis and adenocarcinoma	Rhesus
Helicobacter winghamensis	WP_006803026	67	Gastroenteritis	Humans
Helicobacter trogontu	KGL33062	69	Ulcerative Typhlocolitis	Mice
Sulfurospirillum deleyianum	WP_012856842	69	Nitrite reduction	None
Campylobacter lari	WP_012661337	69	Joint infection and Bacteremia	Humans
Helicobacter bilis	WP_005219380	69	Inflammatory bowel disease	SCID mice
Helicobacter hepaticus	WP_011116467	69	Chronic hepatitis	Mice

Supplementary Table S7. Organisms with metagenomic FabG like ENR carrying organisms.

Organism	Accession Number	Identity %	Importance of the organism
Melioribacter roseus	WP_014854970	66	Cellulytic bacteria
Sphaerobacter thermophilus	YP_003319251	56	Bioremediation
Ignavibacterium album JCM 16511	YP_005845733	57	Chemoheterotroph
Thermomicrobium roseum	YP_002521522	57	Thermophilic bacterium
Deferrisoma camini	YP_002521522	56	Iron(III)-reducing bacterium
Candidatus Methylomirabilis oxyfera	YP_003207365	53	Methane-oxidizing bacterium
Caldithrix abyssi	WP_006928872	52	Nitrate reducing bacterium
Candidatus Solibacter usitatus	YP_823431	51	Thermophilic bacterium
Melioribacter roseus	YP_006526546	52	Thermophilic cellulolytic bacterium
Nitrolancea hollandica	WP_008476078	51	Nitrite-oxidizing bacterium
Streptomyces bingchenggensis	YP_004965000	50	Production of milbemycins and nanchangmycin
Streptomyces violaceusnige	YP_004817797	47	Used as a biocontrol agent
Streptomyces hygroscopicus	WP_030839918	48	Production of rapamycin (Sirolimus) drug
Streptomyces globisporus	WP_010058228	50	Production of the Enediyne antitumor antibiotic C-1027
Streptomyces anulatus	WP_030590816	48	Production of Telomestatin and Endophenazines A-D
Streptomyces floridae	WP_030340953	50	Production of antimicrobial Viomycin
Streptomyces flavotricini	WP_030032568	45	Production of Bafilomycin K, a new antifungal macrolide
Streptomyces avermitilis	NP_824835	45	Production of Avermectins and abamectin
Salinispora arenicola	WP_018809491	44	Produce salinosporamide, a potential anti-cancer agent

Supplementary Table S8. Metagenomic triclosan resistant clones with no known ENR homologues.

**No.**	**Clone**	**ENR/known TCS^{R} Gene candidate**	**Efflux pumps**
1	pBE3	-	1. OM Efflux<br>2. MFS^{T}<br>3. MFS^{T}
2	pAY4-1	-	ABC transporters
3	pAW1	-	-
4	pAX1	-	-
5	pAP3	-	MFS^{T}
6	pAG2	-	1. MFS^{T}<br>2. Hydrolase
7	pW-1	-	ABC transporters
8	pAH4	Hypothetical protein like Novel ENR candidate	ABC transporter
9	pBB2-1	*acrB* (partial)	*acrB* (partial)

Symbols and Abberiviations: -; absent, TCS^{R}; Triclosan resistant, MFS^{T}; MFS transporter, OM; Outer membrane, *acrB*; Acriflavine resistance protein B.

Supplementary Table S9. List of open reading frames from a metagenomic clone, pBE3, and closely related proteins in GenBank.

ORF	No. of amino acids	Closely related proteins (organisms)	% Identity
1 [a]	37	Transposase [*Escherichia coli*]	100
2 [b]	349	Putative outer membrane efflux protein [*Nitrospira defluvii*]	97
3	160	Hypothetical protein [*Nitrospira defluvii*]	96
4	62	Hypothetical protein [*Nitrospira defluvii*]	24
5	357	Glycosyl transferase [*Nitrospira defluvii*]	98
6 [c]	426	MFS transporter [*Nitrospira defluvii*]	98
7	323	Hypothetical protein [*Nitrospira defluvii*]	93
8 [d]	441	MFS transporter [*Nitrospira defluvii*]	97
9	730	Amylo-alpha-1,6-glucosidase [*Nitrospira defluvii*]	97
10	546	Putative peptidase [*Nitrospira defluvii*]	82
11	145	Hypothetical protein [*Nitrospira defluvii*]	92
12	81	Hypothetical protein [*Nitrospira defluvii*]	94
13	276	Hypothetical protein [*Nitrospira defluvii*]	82
14	99	RNA-binding protein [uncultured bacterium A1Q1_fos_560]	98
15	450	ATP-dependent RNA helicase [*Nitrospira defluvii*]	97
16	177	Putative FKBP-type peptidyl-prolyl cis-trans isomerase SlyD [*Nitrospira defluvii*]	97
17 [e]	421	Transposase [*Nitrospira defluvii*]	96
18	316	Putative aldose 1-epimerase [*Nitrospira defluvii*]	93
19	587	Hypothetical protein [*Nitrospira defluvii*]	84
20	176	Hypothetical protein [*Nitrospira defluvii*]	88
21	339	UDP-glucose 4-epimerase [*Nitrospira defluvii*]	92
22	406	Hypothetical protein [*Nitrospira defluvii*]	75
23	43	Hypothetical protein [*Nitrospira defluvii*]	50
24	297	GTP-binding protein YchF [*Nitrospira defluvii*]	95

[a,e] *orf*1,17 encodes mobile elements signatures.[b, c, d] *orf*2, 6, 8 encodes efflux pumps, which might be responsible for the observed resistance to other antibiotics.

Supplementary Table S10. List of open reading frames from a metagenomic clone, pAY4-1, and closely related proteins in GenBank.

ORF	No. of amino acids	Closely related proteins (organisms)	% Identity
1 [a]	208	ABC transporter ATP-binding protein [*Geobacter pickeringii*]	58
2	312	Periplasmic solute binding protein [*Geobacter uraniireducens*]	54
3	143	Fur family transcriptional regulator [*Geobacter uraniireducens*]	58
4	498	Histidine kinase [*Desulfuromonas* sp. TF]	43
5	146	Chemotaxis protein CheY [*Desulfurivibrio alkaliphilus*]	71
6	484	Histidine kinase [delta *proteobacterium* MLMS-1]	49
7 [b]	326	ABC transporter, [*Candidatus Methanoperedens nitroreducens*]	49
8	66	Hypothetical protein [*Desulfuromonas* sp. TF]	25
9	452	DNA repair protein RadA [*Desulfuromonas* sp. TF]	80
10	166	Molybdenum cofactor biosynthesis protein MoaC [*Desulfuromonas* sp. TF]	74
11	169	Hypothetical protein [*Desulfuromonas* sp. TF]	42
12	118	Conjugal transfer protein TraR [*Desulfuromonas* sp. TF]	83
13	445	Histidine kinase [*Desulfuromonas* sp. TF]	41
15	146	Hypothetical protein [*Desulfuromonas* sp. TF]	56
16	624	B12-binding domain-containing radical SAM protein [*Geobacter* sp. OR-1]	52
17	66	Cold-shock protein [*Desulfuromonas* sp. TF]	89
18	267	Hypothetical protein [*Geobacter lovleyi*]	85
19	365	Peptidase M48 Ste24p [*Geobacter lovleyi*]	52
20	84	Hypothetical protein [*Geoalkalibacter subterraneus*]	44
21	59	Hypothetical protein [*Desulfuromonas* sp. WTL]	73
22 [c]	407	ABC transporter permease [*Desulfocurvus vexinensis*]	60
23 [d]	416	ABC-type antimicrobial peptide transport system, permease component [*Desulfovibrio africanus*]	65
24 [e]	225	Macrolide ABC transporter ATP-binding protein [*Pelobacter carbinolicus*]	71
25 [f]	56	Putative transposase [*Escherichia coli*]	100

[a, b, c, d, e] *orf*1, 7, 22, 23, 24 encodes various efflux pumps protein homologues. [f] *orf*,25 encodes mobile elements signatures homologues.

Supplementary Table S11. List of open reading frames from a metagenomic clone, pAW1, and closely related proteins in GenBank.

ORF	No. of amino acids	Closely related proteins (organisms)	% Identity
1 [a]	202	Cytochrome D ubiquinol oxidase subunit I (*Ignavibacterium album*)	61
2 [b]	338	Cytochrome D oxidase subunit I (*Ignavibacterium album*)	64
3	163	Putative metal-dependent hydrolase YfiT (*Candidatus Scalindua brodae*)	47
4 [c]	451	Deoxyribodipyrimidine photo-lyase (candidate division TM6 bacterium GW2011_GWF2_36_6)	50
5	15	tRNA-Glu (*Candidatus Scalindua brodae*)	36
6	165	Phenylacetic acid degradation protein (*Lysobacter dokdonensis*)	41
7	442	Hypothetical protein (*Desulfobulbus japonicus*)	33
8	442	Acetoacetate metabolism regulatory protein AtoC (*Desulfotomaculum kuznetsovii*)	50
9	346	PREDICTED: filamin-A-interacting protein 1 isoform X2 (*Larimichthys crocea*)	22
10	419	Elongator complex protein 1 isoform 2 (*Galdieria sulphuraria*)	32
11	56	Hypothetical protein	29
12	858	FG-GAP repeat protein (candidate division *Zixibacteria bacterium* RBG-1)	31
13	239	MULTISPECIES: porin (*Hyphomicrobium*)	25
14	210	Membrane protein (*Sphingobium herbicidovorans*)	32
15	246	Hypothetical protein (*Chlorobaculum tepidum*)	40
16	210	LemA family protein (*Butyricimonas synergistica*)	44
17	39	Hypothetical protein	34
18	487	Hypothetical protein (*Hydrocarboniphaga effusa*)	48
19	182	Hypothetical protein (*Geobacillus vulcani*)	25
20	355	UDP-N-acetylglucosamine 2-epimerase (*Prolixibacter bellariivorans*)	58
21	475	Class V aminotransferase (*miscellaneous Crenarchaeota* group archaeon SMTZ-80)	50
22	403	Group 1 glycosyl transferase (*Bacillus vireti*)	33
23	169	Hypothetical protein (*Myxococcus stipitatus*)	37
24	272	Hypothetical protein AMJ73_07090 (candidate division *Zixibacteria bacterium* SM1_73)	48
25	118	Hypothetical protein HISP_01140 (*Haloarcula hispanica* N601)	37
26	323	Platelet-activating factor acetylhydrolase plasma/intracellular isoform II (*Pedosphaera parvula*)	38
27	107	RNA-binding protein (*Ornatilinea apprima*)	67
28	224	Heavy metal translocating P-type ATPase, partial (*uncultured bacterium*)	45
29	328	Hypothetical protein RBG1_1C00001G0462 (candidate division *Zixibacteria bacterium* RBG-1)	42
30	84	Hypothetical protein (*Prochlorothrix hollandica*)	56
31	255	Hypothetical protein AA931_11155 (*Peptococcaceae bacterium 1109*)	39
32	403	Sodium/hydrogen exchanger (*Anaeromyxobacter sp. K*)	49
33	92	Hypothetical protein AMJ41_03200 (candidate division *Zixibacteria bacterium* DG_27)	56
34	134	Hypothetical protein RBG1_1C00001G0145 (candidate division *Zixibacteria bacterium* RBG-1)	34
35	287	ATP synthase subunit A (candidate division WOR_1 *bacterium* DG_54_3)	53
36	78	ATP synthase subunit C (candidate division *Zixibacteria bacterium* DG_27)	68
37	160	ATP synthase subunit b (candidate division *Zixibacteria bacterium* RBG-1)	47
38	182	Hypothetical protein RBG1_1C00001G0149 (candidate division *Zixibacteria bacterium* RBG-1)	38
39	502	ATP synthase subunit alpha (candidate division *Zixibacteria bacterium* RBG-1)	74
40	289	Hypothetical protein AMJ41_03210 (candidate division *Zixibacteria bacterium* DG_27)	51
41	40	Hypothetical protein	26

[a, b] *orf*1,2 encodes oxidases whereas [c] *orf*4 encodes photolyases homologoues.

Supplementary Table S12. List of open reading frames from a metagenomic clone, pAX1, and closely related proteins in GenBank.

ORF	No. of amino acids	Closely related proteins (organisms)	% Identity
1	200	Hypothetical protein AMJ49_05165 (*Parcubacteria bacterium* DG_74_2)	47
2	79	Hypothetical protein (*Paenibacillus* sp. DMB20)	49
3	794	Phosphoenolpyruvate synthase (*Leptolinea tardivitalis*)	56
4	377	Hypothetical protein ADN00_01570 (Ornatilinea apprima)	66
5	66	Hypothetical protein (*Calothrix* sp. PCC 7103)	52
6	53	Hypothetical protein (*Calothrix* sp. PCC 7103)	58
7	111	Hypothetical protein (*Coleofasciculus chthonoplastes*)	36
8	640	Hypothetical protein LARV_03539 (*Longilinea arvoryzae*)	39
9	158	Lprotein containing umazine-binding domain (*Leptolinea tardivitalis*)	41
10	600	Oligoendopeptidase, pepF/M3 family (*Anaerolinea thermolimosa*)	60
11	98	Hypothetical protein (*Anaerolinea thermophile*)	51
12	132	Glyoxalase (*Phyllobacterium* sp. YR531)	60
13	80	Cupin (*Ardenticatena maritima*)	59
14	391	Acyl-CoA dehydrogenase (*Leeia oryzae*)	80
15	428	Glutamate dehydrogenase (*Anaerolinea thermophila*)	77
16	255	Hypothetical protein AMJ88_12270 (*Anaerolineae bacterium* SM23_ 63)	48
17	152	Hypothetical protein LTAR_01880 (*Leptolinea tardivitalis*)	67
18	366	Dihydroorotate oxidase A (*Longilinea arvoryzae*)	50
19	516	Hypothetical protein (*Ktedonobacter racemifer*)	23
20	306	GCN5 family acetyltransferase [*Clostridiales bacterium* mt11]	37
21	260	Alpha/beta hydrolase (*Leptospira inadai*)	39
22	244	Metallophosphoesterase [*Sphaerochaeta globosa*]	41
23	182	DNA mismatch repair protein MutT [*Clostridium botulinum*]	41
24	204	Orotate phosphoribosyltransferase [*Longilinea arvoryzae*]	66
25	277	Orotidine-5-phosphate decarboxylase [*Longilinea arvoryzae*]	58
26	1228	Hypothetical protein [*Longilinea arvoryzae*]	33
27	86	Stage V sporulation protein S [*Desulfitobacterium hafniense*]	56
28 [a]	279	Integrase [*Shigella flexneri*]	99
29 [b]	121	Repressor TnpA [*Shigella sonnei*]	100
30	362	Dihydroorotase, multifunctional complex type [*Ktedonobacter racemifer*]	36
31	278	EDD domain protein, DegV family [*Longilinea arvoryzae*]	51
32	254	DNA-binding response regulator [*Roseiflexus* sp. RS-1]	35
33	42	Hypothetical protein	31
34	1223	Hypothetical protein UR29_C0014G0007 [*Microgenomates* (Woesebacteria) bacterium GW2011_GWC2_33_12]	38

[a, b] *orf*28, 29 encodes mobile elements signatures homologues.

Supplementary Table S13. List of open reading frames from a metagenomic clone, pAP3, and closely related proteins in GenBank.

ORF	No. of amino acids	Closely related proteins (organisms)	% Identity
1	213	Hypothetical protein (*Nevskia soli*)	35
2	216	Orotate phosphoribosyltransferase (*Nevskia ramosa*)	71
3	277	DNA-(apurinic or apyrimidinic site) lyase (*Nevskia soli*)	81
4 [a]	466	MFS transporter (*Xanthomonas albilineans*)	70
5	198	GCN5 family acetyltransferase (*Burkholderia ginsengisoli*)	37
6 [b]	344	Aldo/keto reductase (*Nevskia soli*)	73
7	299	Phosphoribulokinase (*Nevskia soli*)	77
8	239	tRNA (guanine-N7)-methyltransferase (*Nevskia soli*)	65
9	297	Thiazole synthase (*Nevskia soli*)	80
10	65	Thiamine biosynthesis protein ThiS (*Marichromatium purpuratum*)	58
11	15	tRNA-Gly	35
12	640	Integrase (*Ralstonia* sp. PBA)	98
13	206	LysR family transcriptional regulator (*Ralstonia* sp. PBA)	88
14	101	MULTISPECIES: transcriptional regulator (*Burkholderiales*)	95
15	632	Integrating conjugative element relaxase (*Ralstonia* sp. PBA)	88
16	210	Hypothetical protein (*Pseudomonas putida*)	75
17	455	Hypothetical protein ALISP_1550 (*Alicycliphilus* sp. B1)	92
18	287	Hypothetical protein DO65_1068 (*Burkholderia pseudomallei*)	96
19	488	Conserved hypothetical protein (*Burkholderia pseudomallei* 406e)	87
20	183	Hypothetical protein (*Achromobacter piechaudii*)	94
21	124	Hypothetical protein (*Pseudomonas aeruginosa*)	69
22	506	Membrane protein (*Ralstonia pickettii*)	91
23	121	Hypothetical protein (*Ralstonia pickettii*)	69
24 [c]	465	Integrating conjugative element protein (*Alicycliphilus denitrificans*)	97
25	220	MULTISPECIES: hypothetical protein (*Proteobacteria*)	97
26	374	Hypothetical protein (*Pectobacterium carotovorum*)	55
27	464	Hypothetical protein (*Pectobacterium carotovorum*)	29
28 [d]	912	Conjugative transfer ATPase (*Pseudomonas aeruginosa*)	96
29	109	Hypothetical protein AzCIB_4459 (*Azoarcus* sp. CIB)	91
30	489	Hypothetical protein (*Pseudomonas aeruginosa*)	95
31 [e]	309	Integrating conjugative element protein, PFL_4704 family (*Alicycliphilus denitrificans K601*)	93
32 [f]	230	Integrating conjugative element protein (*Alicycliphilus denitrificans*)	97
33 [g]	142	MULTISPECIES: conjugative transfer region protein (*Proteobacteria*)	98
34 [h]	124	MULTISPECIES: integrating conjugative element membrane protein (*Proteobacteria*)	90
35	38	MULTISPECIES: hypothetical protein (*Proteobacteria*)	100
36	67	PREDICTED: brorin isoform X3(*Microcebus murinus*)	41
37	128	Hypothetical protein (*Pseudomonas aeruginosa*)	87
38 [i]	114	Integrating conjugative element membrane protein (*Pseudomonas aeruginosa*)	98

[a, b] *orf*4, 6 encodes MFS transporter and aldo-keto reductase respectively. [c, d, e, f, g, h, i, j, k] *orf*24, 28, 31, 32, 33, 34, 38 encodes mobile elements signatures homologues.

Supplementary Table S14. List of open reading frames from a metagenomic clone, pAG2, and closely related proteins in GenBank.

ORF	No. of amino acids	Closely related proteins (organisms)	% Identity
1	42	Ribose-phosphate pyrophosphokinase partial (*Turicella otitidis*)	43
2	258	IclR family transcriptional regulator (*Geobacter sulfurreducens*)	56
3	115	Hypothetical protein (*Geobacter* sp. M21)	84
4	666	SSS sodium solute transporter superfamily (*Geobacter* sp. M21)	78
5	41	Hypothetical protein (*Paramecium tetraurelia* strain d4-2)	70
6	885	Pyruvate phosphate dikinase (*Geobacter lovleyi*)	85
7 [a]	432	MFS transporter (*Geobacter daltonii*)	87
8	394	Hypothetical protein Geob_2856 (*Geobacter daltonii* FRC-32)	54
9	430	Hypothetical protein (*Geobacter* sp. OR-1)	66
10	481	DNA helicase (*Geobacter daltonii*)	76
11	401	Peptidase U32 (*Geobacter* sp. OR-1)	67
12	303	Hypothetical protein (*Geobacter* sp. OR-1)	45
13	288	Diacylglycerol kinase (*Geobacter sulfurreducens*)	41
14	539	Lipid A phosphoethanolamine transferase (*Pelobacter carbinolicus*)	50
15	90	Type I citrate synthase (*Geobacter lovleyi*)	99

[a], *orf* 6 encodes MFS transporter.

Supplementary Table S15. List of open reading frames from a metagenomic clone, pW-1, and closely related proteins in GenBank.

ORF	No. of amino acids	Closely related protein (organisms)	% Identity
1	150	Hypothetical protein ACD_17C00009G0002 [uncultured bacterium]	43
2	465	Glycyl-tRNA synthetease [*Herpetosiphon aurantiacus*]	69
3 [a]	261	ABC transporter ATPase [*Simkania negevensis*]	69
4 [b]	400	ABC transporter, partial [Simkania negevensis]	29
5	190	Hypothetical protein [*Parachlamydia acanthamoebae*]	68
6	372	Hypothetical protein [*Simkania negevensis*]	42
7	128	Hypothetical protein [*Simkania negevensis*]	40

[a, b] *orf3*,4 encodes ABC transporters like homologues, and the DNA sequence determined in pW-1 was partial.

Supplementary Table S16. List of open reading frames from a metagenomic clone, pBB2-1, and closely related proteins in GenBank.

ORF	No. of amino acids	Closely related protein (organisms)	% Identity
1 [a]	159	Acriflavine resistance protein B [*Tolumonas* sp. BRL6-1]	52
2	335	Hypothetical protein (Partial) [*Simkania negevensis*]-Partial	32

[a] *orf*2 encodes *acrB* protein homologue, and the DNA sequence determined in pBB2-1 revealed that this *orf* was covering less than 1/5th of the original protein.

Supplementary Table S17. List of open reading frames from a metagenomic clone, pAH4, and closely related proteins in GenBank.

**ORF**	**No. of amino acids**	**Closely related protein (organisms)**	**% Identity**
1	139	Peptide methionine sulfoxide reductase [*Thiomicrospira crunogena*]	82
2	197	Ferritin [*Herbaspirillum lusitanum*]	63
3	251	Spermidine synthase [*Sulfuricella denitrificans*]	73
4 [a]	619	ABC transporter [*Sulfuricella denitrificans*]	84
5	173	Smr protein/MutS2 [*Sideroxydans lithotrophicus*]	70
6	372	Thioredoxin reductase [*Sideroxydans lithotrophicus*]	90
7	319	Lytic murein transglycosylase [*Sideroxydans lithotrophicus*]	67
8	150	Peptidase [*Sulfuritalea hydrogenivorans*]	55
9	137	30S ribosomal protein S6 [*Sideroxydans lithotrophicus*]	94
10	149	50S ribosomal protein L9 [*Sideroxydans lithotrophicus*]	90
11	472	DNA helicase [*Sideroxydans lithotrophicus*]	88
12	435	Threonyl-tRNA synthetase [*Sideroxydans lithotrophicus*]	93
13	165	Translation initiation factor IF-3 [*Sideroxydans lithotrophicus* ES-1]	87
14	282	Phenylalanyl-tRNA synthetase subunit alpha [*Sideroxydans lithotrophicus*]	86
15	287	Phenylalanyl-tRNA synthetase subunit beta [*Sideroxydans lithotrophicus*]	77
16	250	Stationary phase survival protein SurE, partial [*Sideroxydans lithotrophicus*]	82
17	527	Protein-L-isoaspartate O-methyltransferase [*Burkholderia ambifaria* MEX-5]	59
18	308	Peptidase M23 [*Sideroxydans lithotrophicus*]	45
19	201	Radical SAM protein [*Sideroxydans lithotrophicus*]	72
20	634	Prohead protease [*Sideroxydans lithotrophicus*]	82
21	236	Exonuclease [*Gallionella capsiferriformans*]	82
22	766	Sodium transporter [*Sulfuricella* sp. T08]	85
23	318	Sel1 domain protein repeat-containing protein [*alpha proteobacterium* BAL199]	53
24	381	Swarming motility regulation sensor protein RssA [*Ferriphaselus amnicola*]	64
25	260	Transcriptional regulator [*Sideroxydans lithotrophicus*]	88
26	699	Sodium:solute symporter [*Sideroxydans lithotrophicus*]	80
27	143	PEP synthetase regulatory protein [*Thauera* sp. 63]	65
28	508	Aminoacyl-histidine dipeptidase [*Sulfuricella denitrificans* skB26]	73
29 [b]	439	Hypothetical protein Slit_2509 [*Sideroxydans lithotrophicus* ES-1]	89
30	63	BolA family transcriptional regulator [*Sideroxydans lithotrophicus*]	70
31	104	Glutaredoxin [*Sulfuricella denitrificans*]	76
32	98	Sulfite reductase subunit gamma [*Sideroxydans lithotrophicus*]	80
33	140	Hypothetical protein [*Sideroxydans lithotrophicus*]	51
34	542	Hypothetical protein [*Gallionella capsiferriformans*]	62

[a] *orf*4 encodes ABC transporter.[b] *orf*29 encodes a hypothetical protein homologue, which conferred resistance to triclosan and complemented ENR activity *in vivo*.

Supplementary Table S18. Occurrence of metagenomic pAH4-4 hypothetical protein homologues in other organisms.

Organism	Accession number	% ID	Importance
[α]*Sideroxydans lithotrophicus* ES-1	WP_013030632	89	Iron-oxidizing lithotroph
[α]*Gallionella capsiferriformans* ES-2	WP_013293988	81	Iron-oxidizing lithotroph
[β]*Janthinobacterium agaricidamnosum*	WP_038488551	77	Soft rot pathogen of *Agaricus bisporus*
[α]*Rhodoferax ferrireducens* T118	WP_011462500	75	Oxidizes acetate with the reduction of Fe(III)
[μ]*Massilia timonae*	WP_005666707	71	Wound infections in humans
[□]Rhizobium sp. CF394	WP_037137633	70	Able to fix nitrogen
[□]*Sinorhizobium medicae* WSM419	WP_011970100	70	In association with medicago plants
[β]*Agrobacterium tumefaciens*	WP_042616782	69	Crown gall disease in over 140 species of plants
[□]*Sinorhizobium sp*	WP_037390082	68	Nitrogen fixation
[*]*Ensifer adhaerens*	WP_025430578	67	A bacterial predator of bacteria
[□]*Sinorhizobium meliloti*	WP_027990355	67	Nitrogen fixation, nodulation
[□]*Agrobacterium* sp. H13-3 (on plasmid pAspH13-3a,)	WP_013637535	66	Nonpathogenic, model organism for scientific studies
[□]*Neorhizobium galegae*	WP_041365593	66	Induce nodules on Galega plants
[β]*Agrobacterium vitis*	WP_015917294	65	Crown gall disease of grape
[α]*Ruegeria* sp. R11	WP_008561783	65	Acylhomoserine lactone producing symbiont
[□]*Pseudovibrio* sp. FO-BEG1	WP_014290257	63	Denitrification
[α]*Geobacter sulfurreducens* PCA	WP_010941665	53	Hydrogen and acetate-oxidizing metal-reduction
[α]Geobacter sp. M18	WP_015720969	50	Bioremediation
[α]*Pelobacter propionicus* DSM 2379.	WP_011736501	50	Ethanol fermentation
[α]*Geobacter daltonii* FRC-32	WP_012646832	50	Fe(III)- and uranium(VI)-reducing bacterium
[α]*Geobacter uraniireducens* Rf4	WP_011937857	50	Uranium bioremediation
[α]*Geobacter bemidjiensis Bem*	WP_012531233	48	Iron reduction
[*]*Bdellovibrio sp*. ArHS	WP_041873392	48	A bacterial predator of bacteria

Symbols: *μ*, bacteria isolated from immunocompromised patients; *α,* bacteria related to biogeochemical cycles**;** *β*, bacteria pathogenic to plants or fungi; □, bacteria that are involve in plant symbiosis, nodulation and nitrogen fixation; *****, bacteria which can attack, penetrate and grow in the intraperiplasmic space of other bacteria.

Supplementary Table S19. Table representing various antibiotics which were tested for co/cross resistance.

**No.**	**Antibiotc**	**Major class**
1	Ampicillin	*B-Lactam*
2	Penicillin G	*B-Lactam*
3	Kanamycin	Aminoglycoside
4	Streptomycin	Aminoglycoside
5	Amikacin	Aminoglycoside
6	Sisomicin Sulfate	Aminoglycoside
7	Tobramycin Sulfate	Aminoglycoside
8	Neomycin Sulfate	Aminoglycoside
9	Gentamicin	Aminoglycoside
10	Hygromycin B	Aminoglycoside
11	Tetracycline	Polyketide antibiotic
12	Cefotaxime	Cephalosporins
13	Cefdinir	Cephalosporins
14	Bacitracin	Cell wall inhibitors
15	Polymyxin B	Cell wall inhibitors
16	Spectinomycin	Aminocyclitol class
17	Trimethoprim	Folic acid synthesis inhibitors
18	Chloramphenicol	Amphenicol
19	Florifenicol	Synthetic chloramphenicol derivative

Supplementary Table S20. Co/ cross resistance of metagenomic triclosan resistant clones to other antibiotics.

**Metagenomic clone/organism**	**MIC (µg/ml) to different antibiotics**								
	**Str**	**Gen**	**Kan**	**Neo**	**Spc**	**Tet**	**PolB**	**Cp**	**Amp**
DH5α (pUC119)	45	4	3.5	10	21	5	0.5	7.5	x
DH5α (pUC119)*	45	4	3.5	10	21	5	0.5	7.5	x
EPI-300 (pCC1FOS)	45	4	3.5	10	21	5	0.5	x	4
EPI-300 (pCC1FOS)*	45	4	3.5	10	21	5	0.5	x	4
pAF1-5	100	-	10	21	-	-	-	10	x
pAF1-5*	100	-	10	21	-	-	-	10	x
pAQ2-2	100	10	-	-	-	10	-	-	x
pAQ2-2*	100	10	-	-	-	10	-	-	x
pAV2	100	-	8	-	25	-	-	x	20
pAV2*	100	-	8	-	25	-	-	x	20
pL-1	80	15	-	45	-	-	-	x	-
pL-1*	80	15	-	45	-	-	-	x	-
pAH4	-	6	-	-	-	-	1	x	-
pAH4*	-	6	-	-	-	-	1	x	-
pB-1-4	-	-	-	-	-	-	-	10	x
pB-1-4*	-	-	-	-	-	-	-	10	x
pBE3	-	-	-	45	-	10	-	x	-
pBE3*	-	-	-	45	-	10	-	x	-
pK2	85	-	-	30	-	-	-	x	-
pK2*	85	-	-	30	-	-	-	x	-
pBC1	-	-	-	-	-	15	-	x	-
pBC1*	-	-	-	-	-	20	-	x	-
pAW1	-	-	-	25	-	-	-	x	-
pAW1*	-	-	-	25	-	-	-	x	-
pAP3	-	-	-	20	-	-	-	x	-
pAP3*	-	-	-	20	-	-	-	x	-

Symbols and Abbreviations, Str, Streptomycin; Gen, Gentamicin; Kan, Kanamycin; Neo, Neomycin Sulfate; Spc, Spectinomycin; Tet, Tetracycline; PolB, Polymexin B; Cp, Chloramphenicol; Amp, Ampicillin; *, These subclones were treated with sub-lethal concentrations (0.1 µg/ml) of triclosan prior to check for cross resistance to other antibiotics; -, did not show any resistance; x, not checked for these antibiotics, as the cloning vector contained a gene for resistance to corresponding antibiotic.

Supplementary Table S21. List of open reading frames from a metagenomic clone, pAF1-5, and closely related proteins in GenBank.

**ORF**	**No. of amino acids**	**Closely related protein (organisms)**	**% Identity**
1	49	OsmC family protein [*Pedobacter heparinus* DSM 2366]	61
2 [a]	301	Enoyl-(acyl carrier protein) reductase [*Simkania negevensis* Z]	73
3	134	NUDIX hydrolase [*Holospora undulata*]	55
4	264	Hypothetical protein LPO_1977 [*Legionella pneumophila* subsp. *pneumophila*]	34
5	382	Hypothetical protein SNE_A10980 [*Simkania negevensis* Z]	31
6	124	Exopolysaccharide synthesis protein [*Nostoc* sp. PCC 7120]	36

[a] *orf*2 encodes metagenomic triclosan tolerant YX7K type ENR.

Supplementary Table S22. List of open reading frames from a metagenomic clone, pAQ2-2, and closely related proteins in GenBank.

ORF	No. of amino acids	Closely related protein (organisms)	% Identity
1 [a]	325	Trans-2-enoyl-coA reductase [marine gamma *proteobacterium* HTCC2148]	85
2 [b]	279	Beta-lactamase [marine gamma *proteobacterium* HTCC2148]	69
3	597	Amino-acid metabolism [*Mycobacterium* sp. 012931]	30

[a] *orf*1 encodes metagenomic mild triclosan tolerant FabV ENR homologue, with compromised ENR activity.
[b] *orf*2 encodes metagenomics Beta-lactamase which conferred co resistance to other antibiotics.

Supplementary Table S23. List of open reading frames from a metagenomic clone, pAV2, and closely related proteins in GenBank.

ORF	No. of amino acids	Closely related proteins (organisms)	% Identity
1	53	Cyclopropane fatty acyl phospholipid synthase [*Chlorobium luteolum* DSM 273	71
2	1021	Uncharacterized iron-regulated protein [*Desulfocapsa sulfexigens* DSM 10523	44
3	570	Mg-protoporphyrin IX monomethyl ester oxidative cyclase [*Desulfotalea psychrophila* LSv54]	54
4	394	Phosphoglycerate dehydrogenase-like oxidoreductase [*Desulfocapsa sulfexigens* DSM 10523]	70
5	317	Iron-sulfur cluster-binding protein [*Synechocystis* sp. PCC 6714]	32
6 [a]	261	Short-chain dehydrogenase/reductase SDR [*Desulfobulbus propionicus* DSM 2032]	60
7 [b]	384	RND transporter [*Thiothrix nivea*]	60
8 [c]	187	Antimicrobial peptide ABC transporter ATPase [*Thioflavicoccus mobilis* 8321]	63
9 [d]	402	Antimicrobial peptide ABC transporter permease [*Thioflavicoccus mobilis* 8321]	62
10 [e]	400	ABC transporter permease [*Psychromonas ingrahamii* 37]	56
11 [f]	149	N-acetyltransferase GCN5 [*Runella slithyformis* DSM 19594]	59
12	936	Histidine kinase,response regulator receiver domain, GAF domain-containing protein [*Desulfotignum phosphitoxidans*	62
13	330	Hypothetical protein [*Desulfocapsa sulfexigens*]	33
14	220	5-AMP-activated protein kinase beta-1 subunit [*Desulfobulbus propionicus*]	52
15	186	ECF subfamily RNA polymerase sigma-24 factor [*Desulfobulbus propionicus*]	51
16	143	Osmotically inducible protein C [*Paludibacter propionicigenes*]	49
17	221	Cytochrome C peroxidase [*Desulfobacter postgatei*]	54
18	813	Ntr family sensory/regulatory protein [*Desulfonatronum thiodismutans*]	32
19	274	ABC transporter substrate-binding protein	37
20	170	Hypothetical protein [*Desulfobulbus* sp. Tol-SR]	84
21	234	Hypothetical protein [*Desulfobulbus* sp. Tol-SR]	71
22	169	Hypothetical protein [*Desulfobulbus* sp. Tol-SR]	76
23	159	Cys-tRNA(Pro)/Cys-tRNA(Cys) deacylase ybaK [*Glaciecola lipolytica*]	60

[a] *orf*6 encodes metagenomic triclosan resistant FabL like ENR homologue.

[b, c, d, e, f] *orf*7, 8, 9, 10 and 11 encodes a cluster of efflux pumps and aminoglycoside deactivating enzyme, conferred co resistance to other antibiotics.

Supplementary Table S24. List of open reading frames from a metagenomic clone, PL, and closely related proteins in GenBank.

ORF	No. of amino acids	Closely related protein (organisms)	% Identity
1	182	Membrane-associated zinc metalloprotease [*Sulfuricurvum* sp. MLSB]	76
2	184	CDP-diacylglycerol--glycerol-3-phosphate 3-phosphatidyltransferase [*Sulfuricurvum* sp. MLSB]	86
3[a]	255	7-alpha-hydroxysteroid dehydrogenase [uncultured *Sulfuricurvum* sp. RIFRC-1]	96
4	78	Dihydrodipicolinate synthase [*Sulfuricurvum* sp. MLSB]	85
5	215	Dihydrodipicolinate synthase [*Sulfuricurvum kujiense*]	94
6	433	Protease [*Sulfuricurvum kujiense* DSM 16994]	81
7	352	Dihydroorotate dehydrogenase 2 [*Sulfuricurvum* sp. MLSB]	87
8[b]	568	ABC transporter-like protein [*Sulfuricurvum* sp. MLSB]	79
9	465	Cysteinyl-tRNA ligase [*Candidatus Sulfuricurvum* sp. RIFRC-1]	83
10[c]	467	Multidrug transporter MurJ [*Sulfuricurvum* sp. MLSB]	85
11	634	Hypothetical protein [*Sulfuricurvum kujiense*]	61
12	187	Holliday junction DNA helicase RuvA [*Sulfuricurvum* sp. MLSB]	82
13	345	D-alanyl-alanine synthetase A [uncultured *Sulfuricurvum* sp. RIFRC-1]	82
14	243	2-hydroxy-6-oxohepta-2,4-dienoate hydrolase [*Sulfuricurvum* sp. MLSB]	68
15	479	Mur ligase middle domain-containing protein [uncultured *Sulfuricurvum* sp. RIFRC-1]	79
16	132	HIT family hydrolase [uncultured *Sulfuricurvum* sp. RIFRC-1]	46
17	225	Hypothetical protein [uncultured *Sulfuricurvum* sp. RIFRC-1]	75
18	472	Hypothetical protein [uncultured *Sulfuricurvum* sp. RIFRC-1]	84
19	728	ABC transporter ATP-binding and permease component [uncultured *Sulfuricurvum* sp. RIFRC-1]	78
20	445	Secretion protein [uncultured *Sulfuricurvum* sp. RIFRC-1]	49
21	197	Hypothetical protein [uncultured *Sulfuricurvum* sp. RIFRC-1]	31

[a] *orf*3 encodes 7-alpha-hydroxysteroid dehydrogenase like ENR homologue.

[b,c] *orf*8,10 encodes efflux pumps protein homologues, which might be associated with the observed resistance to other antibiotics.

Supplementary Table S25. List of open reading frames from a metagenomic clone, pB-1-4, and closely related proteins in GenBank.

ORF	No. of amino acids	Closely related protein (organisms)	% Identity
1	95	30S ribosomal protein S20 [*Microbulbifer agarilyticus*]	76
2 [a]	395	Trans-2-enoyl-CoA reductase [*Gilvimarinus chinensis*]	78
3 [b]	177	Alpha/beta fold family hydrolase [*Calothrix* sp. PCC 7507]	29
4	916	Family 2 glycosyl transferase [*Pseudomonas denitrificans* ATCC 13867]	51

[a] *orf*2 encodes metagenomic triclosan tolerant FabV ENR homologue.
[b] *orf*3 encodes Alpha/beta fold family hydrolase homologue, which may be responsible for co-resistance to other antibiotics.

Supplementary Table S26. List of open reading frames from a metagenomic clone, pBF1, and closely related proteins in GenBank.

ORF	No. of amino acids	Closely related protein (organisms)	% Identity
1	185	Membrane-associated zinc metalloprotease [*Sulfuricurvum* sp. MLSB]	75
2	185	Cdp-diacylglycerol--glycerol-3-phosphate 3-Phosphatidyltransferase [*Sulfuricurvum* sp. MLSB]	79
3	94	Rele toxin [*Sulfurospirillum* sp. SCADC]	80
4	83	Antitoxin [*Sulfurospirillum* sp. SCADC]	83
5	41	Hypothetical protein [*Sulfurospirillum* sp. SCADC]	27
6 [a]	258	7-alpha-hydroxysteroid dehydrogenase [*Sulfurovum* sp. FS06-10]	95
7	297	Dihydrodipicolinate synthase [*Candidatus Sulfuricurvum* sp. RIFRC-1]	90
8	433	Protease [*Sulfuricurvum kujiense* DSM 16994]	81
9	352	Diguanylate cyclase [*Sulfuricurvum kujiense* DSM 16994]	70
10	360	Glycosyl transferase family protein [*Sulfuricurvum* sp. MLSB]	77
11 [b]	571	ABC transporter-like protein [*Candidatus Sulfuricurvum* sp. RIFRC-1]	82
12	465	Cysteinyl-trna ligase [uncultured *Sulfuricurvum* sp. RIFRC-1]	87
13 [c]	467	Multidrug transporter murj [uncultured *Sulfuricurvum* sp. RIFRC-1]	61
14	631	Hypothetical protein [uncultured *Sulfuricurvum* sp. RIFRC-1]	79
15	187	Holliday junction dna helicase ruva [uncultured *Sulfuricurvum* sp. RIFRC-1]	84
16	345	D-alanyl-alanine synthetase A [uncultured *Sulfuricurvum* sp. RIFRC-1]	83
17 [d]	243	Alpha/beta hydrolase fold protein [*Sulfuricurvum kujiense*]	66
18	479	Mur ligase middle domain-containing protein [*Sulfuricurvum kujiense*]	79
19	103	Histidine triad (hit) protein [*Sulfuricurvum kujiense*]	79
20	217	Hypothetical protein [*Sulfuricurvum kujiense*]	46
21	466	Hypothetical protein [*Sulfuricurvum kujiense*]	70
22 [e]	726	ABC transporter atp-binding and permease component [*Arcobacter butzleri*]	84
23	445	Secretion protein (*acra*) [*Arcobacter butzleri*]	81
24	197	Two-component response regulator [*Campylobacter curvus*]	56

[a] *orf*6 encodes 7-alpha-hydroxysteroid dehydrogenase like ENR homologue.
[b,c,d,e] *orf*11,13,17,22 encodes various efflux pumps protein homologues or hydrolases, which might be associated with the observed resistance to other antibiotics.

Supplementary Table S27. List of open reading frames from a metagenomic clone, pBC1, and closely related proteins in GenBank.

ORF	No. of amino acids	Closely related protein (organisms)	% Identity
1	478	Hypothetical protein [*Enterobacter cloacae*]	41
2	603	Hypothetical protein [*Sulfuricurvum kujiense*]	75
3 [a]	569	Hypothetical protein (*acrB*) [*Sulfuricurvum* sp. MLSB]	88
4 [b]	529	Hypothetical protein (*acrB*) [*Sulfuricurvum* sp. MLSB]	94
5	261	Hypothetical protein [uncultured *Sulfuricurvum* sp. RIFRC-1]	96
6 [c]	454	RND transporter [*Pseudomonas aeruginosa*]	15
7	188	Hypothetical protein [*Fictibacillus gelatini*]	44
8	188	4-methyl-5-thiazole monophosphate biosynthesis protein [*Sulfurimonas autotrophica*]	69
9	248	Hypothetical protein [*Sulfuricurvum kujiense*]	58
10	110	Flagellar protein flag protein [*Sulfuricurvum kujiense*]	64
11	642	Signal transduction histidine kinase [*Sulfuricurvum kujiense*]	68
12	231	Winged helix family two component transcriptional regulator [*Desulfurispora thermophila*]	25
13	84	Hypothetical protein [*Sulfuricurvum kujiense*]	75
14	148	Hypothetical protein [*Photobacterium ganghwense*]	45
15	1184	DNA-dirted DNA polymerase [*Sulfuricurvum kujiense* DSM 16994]	94
16	360	Hypothetical protein [*Levilinea saccharolytica*]	51
17	206	Hypothetical protein [*Syntrophomonas wolfei*]	12
18	87	Antitoxin YefM [*Escherichia coli*]	20
19	284	DNA-damage-inducible protein D family protein [*Chlorobium phaeobacteroides*]	53
20	412	Aminopeptidase [*Pseudomonas oryzihabitans*]	48
21	328	Histidine kinase [*Sulfuricurvum kujiense* DSM 16994]	89
22	736	Diguanylate cyclase/phosphodiesterase [uncultured *Sulfuricurvum* sp. RIFRC-1]	80
23	224	Alanine racemase domain-containing protein [*Methylococcus capsulatus*]	38
24	350	Membrane-associated zinc metalloprotease [uncultured *Sulfuricurvum* sp. RIFRC-1]	92
25	202	CDP-diacylglycerol--glycerol-3-phosphate 3-Phosphatidyltransferase [*Sulfurimonas gotlandica*]	74
26 [d]	258	7-alpha-hydroxysteroid dehydrogenase [*Clostridium sordellii*]	35
27	297	Dihydrodipicolinate synthase [*Nitrospina gracilis*]	47
28	433	Protease [*Sulfuricurvum kujiense* DSM 16994]	90
29	352	Dihydroorotate dehydrogenase 2 [uncultured *Sulfuricurvum* sp. RIFRC-1]	99
30 [e]	549	ABC transporter-like protein [*Sulfuricurvum* sp. MLSB]	83
31	422	Cysteinyl-tRNA synthetase [*Escherichia coli*]	40

[a,b,c,e] *orf*3,4,6,30 encodes various efflux pumps protein homologues, which might be associated with the observed resistance to other antibiotics. [d] *orf*26 encodes 7-alpha-hydroxysteroid dehydrogenase like ENR homologue.

Supplementary Table S28. The co-localized antibiotic resistance genes in metagenomic triclosan resistant clones.

No.	Subclone	Known Ab^R genes	Efflux pumps	ENRs
1	pAF1-5	-	-	-
2	pAQ2-2	Beta-lactamase	-	FabV
3	pAV2	N-acetyltransferase	1. ABC transporter<br>2. RND Protein	FabL
4	pL1	-	1. ABC transporter<br>2. MurJ	7-α-HSDH
5	pAH4	-	ABC transporter	Hypothetical protein like ENR candidate
6	pB-1-4	-	-	-
7	pBE3	-	1. OM Efflux<br>2. MFS^T<br>3. MFS^T	-
8	pBF1-4	-	1. MurJ<br>2.ABC transporters<br>3. *acrA*	7-α-HSDH
9	pBC1	-	1. *acrB*<br>2. *acrB*<br>3. ABC transporter	7-α-HSDH
10	pAW1	-	-	-
11	pAP3	-	MFS^T	-

Symbols and Abberiviations: -; absent, Ab^R; Antibiotic resistant, MFS^T; MFS transporter, OM; Outer membrane, RND; Resistance nodulation division, *acrB*; Acriflavin resistance protein B, *acrA*; Acriflavin resistance protein A, 7-α-HSDH; 7-α-hydroxysteroid dehydrogenase.

Supplementary Table S29. Presence of mobile elements signatures in metagenomic triclosan resistant clones.

No.	Clone	Mobile/transposable element
1	pBE3	Present
2	pAY4-1	Present
3	pAY4-2	Present
4	pAX1	Present
5	pAP3	Present
6	pY-3	Present
7	pAU2-3	Present

Supplementary Table S30. Metagenomic clones/subclones with Genbank accession numbers.

No.	Clone Name	GenBank accession numbers
1	pAB1-2	KT860427
2	pAF1-5	KT860426
3	pBB2-1	KT860425
4	pAQ2-2	KT860430
5	pAT4-2	KT860431
6	pJ-1-4	KT860432
7	pQ-2-2	KT860433
8	pQ2N	KT860434
9	pY-3	KT860435
10	pR-2-2	KT860436
11	pAY2-1	KT860437
12	pX-1	KT860438
13	pW-1	KT860439
14	pB-1-4	KT860440
15	pD-1-6	KT860441
16	pAU2-3	KT868816
17	pAH4	KT884454
18	pAV2	KT884455
19	pA-2	KT860428
20	pAL1-4	KT860429
21	pAXI	KT982357
22	pAG2	KT982358
23	pAP3	KT982359
24	pAW1	KT982360
25	pAY4-1	KT982361
26	pAZ2	KT982362
27	pAM1	KT982363
28	pAY4-2	KT982364
29	pBC1	KT982365
30	pBE3	KT982366
31	pBF1	KT982367
32	pL	KT982368

Supplementary Table S31. Strains and plasmids used in this work.

Strain or plasmid/ others	Relevant characteristics[a]	Source or reference
E. coli		
DH5α	F-, *Δ(argF-lac)169*, φ80d*lacZ58*(M15), *ΔphoA8*, *glnX44*(AS), *λ*-, *deoR481*, *rfbC1*?, *gyrA96*(NalR), *recA1*, *endA1*, *thiE1*, *hsdR17*	[Sambrook *et al*., 1989]
EPI-300	F-, *mcrA Δ(mrr-hsd RMS-mcrBC) Φ80dlacZΔM15 ΔlacX74 recA1 endA1 araD139 Δ(ara, leu)7697 galU galK λ- rpsL (StrR) nupG trfA tonA*	[Epicentre]
JP1111	*galE45*(GalS), *λ*-, *fabI392*(ts), *relA1*, *spoT1*, *thiE1*	[Egan AF & Russell RRB, 1973]
CL37	*fabG(*Ts*) fabF::*Kan *zce-727::*Tn*10 of* MG1655	[Lai CY & Cronan JE, 2004]
Plasmids		
pCC1FOS	Cp^{r}, copy control fosmid vector	[Epicentre]
pUC119	Ap^{r}, cloning vector	[Yanisch-Perron, 1985]
pGEM®-T Easy	Ap^{r}, cloning vector	[Promega]
p*Cjej*7α	7-α-HSDH gene of *Campylobacter jejuni* NCTC11168 in pGEM®-T Easy	[This study]
p*Hpy*7α	7-α-HSDH gene of *Helicobacter pylori* HPKTCC B0100 in pGEM®-T Easy	[This study]
Transposon		
EZ-Tn5™ <KAN-2> transposon	Kan^{r}, Random mutagenesis of plasmid DNA	[Epicentre]

[a] Ap^{r}, ampicillin resistance; Cp^{r}, chloramphenicol resistance; Tc^{r}, tetracycline resistance; Kan^{r} , Kanamycin resistance.

References

Egan, A.F. and Russell, R.R.B. (1973) Conditional mutations affecting the cell envelope of *Escherichia coli* K-12. *Genet Res* **21:** 139–152.

Lai, C.Y. and Cronan, J.E. (2004) Isolation and characterization of β-ketoacyl-acyl carrier protein reductase (*fabG*) mutants of *Escherichia coli* and *Salmonella enterica* serovar Typhimurium. *J Bacteriol* **186:** 1869–1878.

Sambrook, J., Fritsch, E. and Maniatis, T. (1989) Molecular Cloning: a Laboratory Manual. Cold Spring Harbor Laboratory Press: New York.

Yanisch-Perron, C., Vieira, J. and Messing, J. (1985) Improved M13 phage cloning vectors and host strains: nucleotide sequences of the M13mpl8 and pUC19 vectors. *Gene* **33:** 103–119.
